# Dataset of volatile compounds identified, quantified and GDA generated of the maturation process of silver tequila in new French oak barrels

**DOI:** 10.1016/j.dib.2019.104707

**Published:** 2019-10-23

**Authors:** S.T. Martín-del-Campo, J.E. López-Ramírez, M. Estarrón-Espinosa

**Affiliations:** a Tecnológico de Monterrey, Escuela de Ingeniería y Ciencias, Centro de Bioingeniería, Epigmenio González 500, Fracc. San Pablo, Querétaro, Qro, 76130, Mexico; b Unidad de Tecnología de Alimentos, Centro de Investigación y Asistencia en Tecnología y Diseño del Estado de Jalisco, A.C., Av. Normalistas 800, Colinas de la Normal, Guadalajara, Jalisco, CP 44270, Mexico

**Keywords:** Tequila, Volatile compound, GC-MS, French oak barrels, GDA analysis

## Abstract

This article contains data obtained by following the evolution of minor volatile compounds throughout 32 weeks of 100% Agave Silver tequila maturation in new French oak barrels under real cellar conditions. Barrels were made with the same cooperage methods in four French regions. Tequila samples were obtained every 2 weeks; volatile compounds were extracted and analyzed by GC-MS. Volatile compounds were identified and relatively quantified in % of Area. Obtained data are presented in three datasets: Identified compounds, quantification according to barrel origin, and quantification according to maturation time. General Discriminant Analysis of the quantification data sets are also shown. Interpretation of the data and discussion can be found in “Evolution of volatile compounds during the maturation process of Silver tequila in new French oak barrels” Martín-del-Campo, López-Ramírez and Estarrón-Espinosa [1].

**Table:** Specifications Table

Subject area	*Food chemistry.*
More specific subject area	*Aroma evolution during Tequila maturation.*
Type of data	*Tables.*
How data was acquired	*GC-MS (HP 5890 Series II coupled to a mass detector HP 5972) with capillary DB-Wax column (Hewlett-Packard). Compounds were tentatively identified with the Wiley 175L spectra library. Identity was confirmed with reference standards and/or by comparison with the Kovats index. Relative quantification was done with the percent area method.*
Data format	*Processed and analyzed.*
Experimental factors	*Tequila sample's volatile compounds were extracted by liquid-liquid extraction (Pentane:Dichloromethane 3:1 v/v) and concentrated in a Kuderna-Danish device.*
Experimental features	*100% Agave silver tequila (just after distillation) was placed in new French oak barrels from four different regions and maturated under real cellar conditions. Samplings were performed from week 2 until week 32 every 2 weeks for each barrel region.*
Data source location	*Tequila region, Jalisco, México.*
Data accessibility	*Data is with this article.*
Related research article	*Evolution of volatile compounds during the maturation process of Silver tequila in new French oak barrels (in press) Martín-del-Campo, López-Ramírez and Estarrón-Espinosa* [1].

**Table:** 

**Value of the Data** • This data shows a comprehensive list of volatile compounds identified in Tequila samples maturated in French oak barrels from four regions and at different maturation times. A total of 173 minor volatile compounds were identified; they belong to different chemical families. This list is the most extensive list of volatile compounds identified in tequila samples throughout the maturation process. • Data sets about volatile compounds quantification according to the barrel origin and maturation provide information about abundance and evolution of the different compounds and chemical families and associate them with the barrel origin and the maturation time. • General discriminant analysis of the volatile compounds quantification data is useful to show the impact of changes in the volatile composition to discriminate samples according to the barrel origin and according to the maturation time.

## 1 Data

Data presented correspond to the minor volatile compounds identified in Tequila samples at different maturation time (weeks) and according with the oak barrels origin region (Table 1) as well as the relative mean concentration of volatile compounds (% area) according with barrels origin (Table 2) and maturation time (Table 3). Additionally, General discriminant analysis classification tables, test of significance of Squared Mahalanobis distances, and statistics for each tequila sample are presented according to barrel origin (Table 4, Table 5, Table 6) and maturation time (Table 7, Table 8, Table 9). All the presented data are related to a research article in press [1].

**Table 1:** Volatile compounds identified, classified according to the maturation time in weeks and the oak barrel origin.

Compound	CAS^a^	Identification^b^	Maturation time (weeks)																	Region^c^			
			0	2	4	6	8	10	12	14	16	18	20	22	24	25	28	30	32	I	II	III	IV
**Acetals**																							
1,1-diethoxy-ethane	105-57-7	MS, RI, ST	✓	✓	✓	✓	✓	✓	✓	✓	✓	✓	✓	✓	✓	✓	✓	✓	✓	✓	✓	✓	✓
1-(1-ethoxyethoxy)pentane	13442-89-2	MS, RI	✓	✓	✓	✓	✓	✓	✓	✓	✓	✓	✓	✓	✓	✓	✓	✓	✓	✓	✓	✓	✓
1,1,1-triethoxyethane	78-39-7	MS, RI			✓	✓	✓	✓	✓	✓	✓	✓	✓	✓	✓	✓	✓	✓	✓	✓	✓	✓	✓
1,1,1-triethoxypropane	115-80-0	MS, RI				✓		✓		✓		✓	✓	✓	✓	✓	✓	✓	✓	✓	✓	✓	✓
1,1,3,3-tetraethoxypropane	122-31-6	MS, RI	✓	✓	✓	✓	✓	✓	✓	✓	✓	✓	✓	✓	✓	✓	✓	✓	✓	✓	✓	✓	✓
1,1-diethoxy-3-methylbutane	3842-03-3	MS, RI	✓	✓	✓	✓	✓	✓	✓	✓	✓	✓	✓	✓	✓	✓	✓	✓	✓	✓	✓	✓	✓
1,1-diethoxyhexane	3658-93-3	MS, RI		✓		✓	✓	✓	✓	✓	✓	✓	✓	✓	✓	✓	✓	✓	✓	✓	✓	✓	✓
1,1-diethoxyisobutane	1741-41-9	MS, RI	✓	✓					✓	✓	✓										✓	✓	✓
1,1-diethoxymethane	462-95-3	MS, RI			✓			✓			✓	✓	✓							✓	✓	✓	✓
1,1-diethoxypropane	4744-08-5	MS, RI, ST				✓	✓	✓	✓	✓	✓	✓	✓	✓	✓	✓	✓	✓	✓	✓	✓	✓	✓
1,1-dimethoxypropane	4744-10-9	MS, RI				✓	✓	✓	✓	✓	✓	✓	✓	✓	✓	✓	✓	✓	✓	✓	✓	✓	✓
1-ethoxy-1-methoxyethane	10471-14-4	MS		✓	✓	✓	✓	✓	✓	✓	✓	✓	✓	✓	✓	✓	✓	✓	✓	✓	✓	✓	✓
1-ethoxy-2-methoxyethane	5137-45-1	MS				✓	✓	✓	✓		✓	✓	✓	✓	✓	✓	✓	✓	✓	✓	✓	✓	✓
2-(diethoxymethyl)furan	13529-27-6	MS, RI									✓	✓	✓		✓	✓	✓	✓	✓	✓	✓	✓	✓
**Acids**																							
2,4,5-trioxoimidazolidine	120-89-8	MS		✓	✓	✓	✓	✓	✓	✓	✓	✓	✓	✓	✓	✓	✓	✓	✓	✓	✓	✓	✓
2-methylbutanoic acid	116-53-0	MS, RI					✓					✓	✓	✓	✓	✓	✓	✓	✓	✓	✓	✓	✓
2-methylpropanoic acid	79-31-2	MS, RI		✓	✓		✓	✓		✓	✓	✓	✓	✓	✓	✓	✓	✓	✓	✓	✓	✓	✓
3-methylbutanoic acid	503-74-2	MS, RI, ST		✓	✓	✓	✓	✓	✓	✓	✓	✓	✓	✓	✓	✓	✓	✓	✓	✓	✓	✓	✓
Benzoic acid, *p*-*tert*-butyl-	98-73-7	MS, RI		✓	✓	✓	✓	✓	✓	✓	✓			✓			✓			✓	✓	✓	✓
Butanoic acid	107-92-6	MS, RI, ST	✓					✓	✓	✓	✓	✓	✓	✓	✓	✓	✓	✓	✓	✓	✓	✓	✓
Decanoic acid	334-48-5	MS, RI, ST	✓	✓	✓	✓	✓	✓	✓	✓	✓	✓	✓	✓	✓	✓	✓	✓	✓	✓	✓	✓	✓
Dodecanoic acid	143-07-7	MS, RI, ST		✓	✓	✓	✓	✓	✓	✓	✓	✓	✓	✓	✓	✓	✓	✓	✓	✓	✓	✓	✓
Ethanoic acid	64-19-7	MS, RI, ST		✓	✓	✓	✓	✓	✓	✓	✓	✓	✓	✓	✓	✓	✓	✓	✓	✓	✓	✓	✓
Hexanoic acid	142-62-1	MS, RI, ST	✓	✓	✓	✓	✓	✓		✓	✓	✓	✓	✓	✓	✓	✓	✓	✓	✓	✓	✓	✓
Hexadecanoic acid	57-10-3	MS, RI, ST		✓	✓	✓	✓	✓	✓	✓	✓	✓	✓	✓	✓	✓	✓	✓	✓	✓	✓	✓	✓
Octanoic acid	124-07-2	MS, RI, ST	✓	✓	✓	✓	✓	✓	✓	✓	✓	✓	✓	✓	✓	✓	✓	✓	✓	✓	✓	✓	✓
Pentanoic acid	109-52-4	MS, RI, ST					✓	✓	✓	✓	✓	✓	✓	✓						✓	✓	✓	✓
Propanoic acid	79-09-4	MS, RI, ST				✓	✓		✓		✓	✓	✓	✓	✓	✓	✓	✓	✓	✓	✓	✓	✓
Tetradecanoic acid	544-63-8	MS, RI, ST	✓	✓	✓	✓	✓	✓	✓	✓	✓	✓	✓	✓	✓	✓	✓	✓	✓	✓	✓	✓	✓
**Alcohols**																							
(4-propan-2-ylphenyl) methanol	536-60-7	MS, RI			✓	✓	✓	✓	✓	✓	✓	✓	✓	✓	✓	✓	✓	✓	✓	✓	✓	✓	✓
1-butanol	71-36-3	MS, RI, ST	✓	✓	✓	✓	✓	✓	✓	✓	✓	✓	✓	✓	✓	✓	✓	✓	✓	✓	✓	✓	✓
1-decanol	112-30-1	MS, RI, ST	✓	✓	✓	✓	✓	✓	✓	✓	✓	✓	✓	✓	✓	✓	✓	✓	✓	✓	✓	✓	✓
1-dodecanol	112-53-8	MS, RI, ST		✓	✓	✓	✓	✓	✓	✓	✓	✓	✓	✓	✓	✓	✓	✓	✓	✓	✓	✓	✓
1-hexadecanol	36653-82-4	MS, RI, ST		✓		✓	✓	✓	✓	✓	✓	✓	✓		✓	✓	✓	✓	✓	✓	✓	✓	✓
1-hexanol	111-27-3	MS, RI, ST		✓	✓	✓	✓	✓	✓	✓	✓	✓	✓	✓	✓	✓	✓	✓	✓	✓	✓	✓	✓
1-pentanol	71-41-0	MS, RI, ST	✓	✓	✓	✓	✓	✓		✓	✓	✓	✓	✓	✓	✓	✓	✓	✓	✓	✓	✓	✓
1-propanol	71-23-8	MS, RI, ST	✓	✓	✓	✓	✓	✓	✓	✓	✓	✓	✓	✓	✓	✓	✓	✓	✓	✓	✓	✓	✓
1-tetradecanol	112-72-1	MS, RI, ST	✓	✓	✓	✓	✓	✓	✓	✓	✓	✓	✓	✓	✓	✓	✓	✓	✓	✓	✓	✓	✓
2-butanol	78-92-2	MS	✓	✓	✓	✓	✓	✓	✓	✓	✓	✓	✓	✓	✓	✓	✓	✓	✓	✓	✓	✓	✓
2-decanol	1120-06-5	MS		✓		✓	✓	✓	✓	✓	✓	✓	✓	✓	✓	✓	✓	✓	✓	✓	✓	✓	✓
2-heptanol	543-49-7	MS, RI		✓	✓	✓	✓	✓	✓	✓	✓	✓	✓	✓	✓	✓	✓	✓	✓	✓	✓	✓	✓
2-hexanol	626-93-7	MS, RI					✓	✓				✓	✓	✓	✓	✓	✓	✓	✓	✓	✓	✓	✓
2-methyl propan-1-ol	78-83-1	MS, RI, ST		✓	✓	✓	✓	✓	✓	✓	✓	✓	✓	✓	✓	✓	✓	✓	✓	✓	✓	✓	✓
2-methyl-3-penten-1-ol	62238-37-3	MS		✓	✓	✓	✓	✓	✓			✓	✓	✓	✓	✓	✓	✓	✓	✓	✓	✓	✓
2-methylbut-2-en-1-ol	4675-87-0	MS		✓	✓	✓	✓	✓	✓	✓	✓	✓	✓	✓	✓	✓	✓	✓	✓	✓	✓	✓	✓
2-nonanol	628-99-9	MS		✓		✓	✓	✓		✓	✓	✓	✓	✓	✓	✓	✓	✓	✓	✓	✓	✓	✓
2-pentanol	6032-29-7	MS, RI				✓	✓	✓	✓		✓	✓	✓	✓	✓	✓	✓	✓	✓	✓	✓	✓	✓
2-phenylethanol	60-12-8	MS, RI, ST		✓	✓	✓	✓	✓	✓	✓	✓	✓	✓	✓	✓	✓	✓	✓	✓	✓	✓	✓	✓
3,4-dimethylpentan-1-ol	6570-87-2	MS, RI	✓			✓	✓	✓	✓	✓	✓	✓	✓	✓	✓	✓	✓	✓	✓	✓	✓	✓	✓
3-ethoxypropan-1-ol	111-35-3	MS, RI	✓	✓	✓	✓	✓	✓	✓	✓	✓	✓	✓	✓	✓	✓	✓	✓	✓	✓	✓	✓	✓
3-methylbut-3-en-1-ol	763-32-6	MS, RI	✓	✓		✓	✓	✓	✓	✓	✓	✓	✓	✓	✓	✓	✓	✓	✓	✓	✓	✓	✓
3-methylbutan-1-ol	123-51-3	MS, RI, ST	✓	✓	✓	✓	✓	✓	✓	✓	✓	✓	✓	✓	✓	✓	✓	✓	✓	✓	✓	✓	✓
3-methylpentan-1-ol	20281-83-8	MS, RI		✓	✓	✓	✓	✓	✓	✓	✓	✓	✓	✓	✓	✓	✓	✓	✓	✓	✓	✓	✓
3-octanol	589-98-0	MS, RI, ST	✓	✓	✓	✓	✓	✓	✓	✓	✓	✓	✓	✓	✓	✓	✓	✓	✓	✓	✓	✓	✓
3-penten-1-ol	764-37-4	MS, RI		✓	✓	✓	✓	✓	✓	✓	✓	✓	✓	✓	✓	✓	✓	✓	✓	✓	✓	✓	✓
3-phenylpropan-1-ol	122-97-4	MS, RI				✓	✓	✓	✓	✓	✓	✓	✓	✓	✓		✓			✓	✓	✓	✓
4-methylpentan-1-ol	68526-79-4	MS, RI		✓	✓	✓	✓	✓	✓	✓	✓	✓	✓	✓	✓	✓	✓	✓	✓	✓	✓	✓	✓
Cyclohex-2-en-1-ol	822-67-3	MS, RI					✓		✓		✓	✓	✓	✓							✓	✓	✓
Oct-2-en-1-ol	22104-78-5	MS, RI	✓		✓			✓		✓	✓									✓	✓	✓	✓
Pent-4-en-1-ol	821-09-0	MS		✓	✓	✓	✓	✓	✓	✓	✓	✓	✓	✓	✓	✓	✓	✓	✓	✓	✓	✓	✓
Phenylmethanol	100-51-6	MS, RI, ST			✓	✓	✓	✓	✓	✓	✓	✓	✓	✓	✓	✓	✓	✓	✓	✓	✓	✓	✓
**Aldehydes**																							
2,6,6-trimethylcyclohexa-1,3-diene-1-carbaldehyde	116-26-7	MS, RI		✓	✓	✓	✓	✓	✓	✓	✓	✓	✓	✓	✓	✓	✓	✓	✓	✓	✓	✓	✓
2,6,6-trimethylcyclohexene-1-carbaldehyde	432-25-7	MS, RI					✓	✓			✓	✓	✓	✓	✓		✓	✓	✓	✓	✓	✓	✓
2-phenylacetaldehyde	122-78-1	MS, RI						✓		✓	✓			✓			✓			✓	✓	✓	✓
3-ethoxypropanal	63918-98-9	MS, RI		✓	✓	✓	✓	✓	✓	✓	✓	✓	✓	✓	✓	✓	✓	✓	✓	✓	✓	✓	✓
3-methylbutanal	590-86-3	MS, RI		✓	✓	✓	✓	✓	✓	✓	✓	✓	✓	✓	✓	✓	✓	✓	✓	✓	✓	✓	✓
4-hydroxy-3,5-dimethoxybenzaldehyde	134-96-3	MS				✓	✓	✓		✓		✓	✓	✓	✓			✓	✓	✓	✓	✓	✓
4-hydroxy-3-methoxybenzaldehyde	121-33-5	MS, RI, ST		✓	✓	✓	✓	✓	✓	✓	✓	✓	✓	✓	✓	✓	✓	✓	✓	✓	✓	✓	✓
4-propan-2-ylcyclohexene-1-carbaldehyde	21391-98-0	MS, RI					✓			✓	✓									✓	✓	✓	✓
Benzaldehyde	100-52-7	MS, RI, ST		✓	✓	✓	✓	✓	✓	✓	✓	✓	✓	✓	✓	✓	✓	✓	✓	✓	✓	✓	✓
Hexanal	66-25-1	MS, RI		✓	✓	✓	✓	✓	✓		✓	✓	✓	✓	✓	✓	✓	✓	✓	✓	✓	✓	✓
**Ketones**																							
(E)-1-(2,6,6-trimethylcyclohexa-1,3-dien-1-yl)but-2-en-1-one	23726-93-4	MS, RI	✓	✓	✓	✓	✓	✓	✓	✓	✓	✓	✓	✓	✓	✓	✓	✓	✓	✓	✓	✓	✓
(E)-4-(2,6,6-trimethylcyclohex-2-en-1-yl)but-3-en-2-one	127-41-3	MS, RI				✓	✓	✓	✓	✓	✓	✓	✓	✓	✓	✓	✓	✓	✓	✓	✓	✓	✓
3-methylcyclopentan-1-one	1757-42-2	MS, RI		✓	✓	✓	✓	✓	✓	✓	✓	✓	✓	✓	✓	✓	✓	✓	✓	✓	✓	✓	✓
Butane-2,3-dione	431-03-8	MS, RI		✓		✓	✓	✓	✓	✓	✓	✓	✓	✓	✓	✓	✓	✓	✓	✓	✓	✓	✓
Cyclopentanone	120-92-3	MS, RI	✓	✓	✓	✓	✓	✓	✓	✓	✓	✓	✓	✓	✓	✓	✓	✓	✓	✓	✓	✓	✓
Hexan-2-one	591-78-6	MS, RI		✓	✓	✓	✓	✓	✓	✓	✓	✓	✓	✓	✓	✓	✓	✓	✓	✓	✓	✓	✓
Pentan-2-one	107-87-9	MS, RI		✓	✓	✓	✓	✓	✓	✓										✓	✓	✓	✓
**Esters**																							
1-phenylpropyl acetate	2114-29-6	MS				✓		✓		✓	✓			✓	✓	✓	✓	✓		✓	✓	✓	
2-methylpropyl acetate	110-19-0	MS, RI		✓	✓	✓	✓	✓	✓	✓	✓	✓	✓	✓	✓	✓	✓	✓	✓	✓	✓	✓	✓
Acetic acid 2-phenylethyl ester	103-45-7	MS, RI		✓	✓	✓	✓	✓	✓	✓	✓	✓	✓	✓	✓	✓	✓	✓	✓	✓	✓	✓	✓
2-phenylethyl propionate	122-70-3	MS, RI		✓		✓	✓	✓		✓	✓	✓	✓	✓	✓	✓	✓	✓	✓	✓	✓	✓	✓
3-methylbutyl acetate	123-92-2	MS, RI	✓	✓	✓	✓	✓	✓	✓	✓	✓	✓	✓	✓	✓	✓	✓	✓	✓	✓	✓	✓	✓
3-methylbutyl decanoate	2306-91-4	MS, RI	✓	✓	✓	✓	✓	✓	✓	✓	✓	✓	✓	✓	✓	✓	✓	✓	✓	✓	✓	✓	✓
3-methylbutyl octanoate	2035-99-6	MS, RI		✓	✓	✓	✓	✓	✓	✓	✓	✓	✓	✓	✓	✓	✓	✓	✓	✓	✓	✓	✓
Benzyl benzoate	120-51-4	MS, RI	✓	✓	✓	✓	✓	✓	✓	✓	✓	✓	✓	✓	✓	✓	✓	✓	✓	✓	✓	✓	✓
bis (2-ethylhexyl) benzene-1,2-dicarboxylate	117-81-7	MS		✓	✓	✓	✓	✓	✓	✓				✓	✓	✓	✓	✓	✓	✓	✓	✓	✓
Dibutyl benzene-1,4-dicarboxylate	1962-75-0	MS, RI		✓		✓	✓	✓	✓	✓	✓			✓	✓	✓	✓	✓	✓	✓	✓	✓	✓
Butanedioic acid, diethyl ester	123-25-1	MS, RI, ST			✓	✓	✓	✓	✓	✓	✓	✓	✓	✓	✓	✓	✓	✓	✓	✓	✓	✓	✓
Ethyl (2S)-2-hydroxypropanoate	2005-07-19	MS			✓	✓	✓	✓	✓	✓	✓	✓	✓	✓	✓	✓	✓	✓	✓	✓	✓	✓	✓
Ethyl (9Z,12Z)-octadeca-9,12-dienoate	544-35-4	MS, RI	✓	✓	✓	✓	✓	✓	✓	✓	✓	✓	✓	✓	✓	✓	✓	✓	✓	✓	✓	✓	✓
Ethyl (9Z,12Z,15Z)-octadeca-9,12,15-trienoate	1191-41-9	MS, RI		✓	✓	✓	✓	✓	✓	✓	✓	✓			✓		✓			✓	✓	✓	✓
Ethyl (Z)-octadec-9-enoate	111-62-6	MS, RI		✓	✓	✓	✓	✓	✓	✓	✓	✓	✓	✓	✓	✓	✓	✓	✓	✓	✓	✓	✓
Ethyl 2-furancarboxylate	614-99-3	MS, RI	✓	✓	✓	✓	✓	✓	✓	✓	✓	✓	✓	✓	✓	✓	✓	✓	✓	✓	✓	✓	✓
Ethyl 2-hydroxy-3-methylbutanoate	2441-06-7	MS, RI		✓		✓	✓	✓	✓	✓	✓	✓	✓	✓	✓	✓	✓	✓	✓	✓	✓	✓	✓
Ethyl 2-hydroxy-propanoate	97-64-3	MS, RI		✓	✓	✓	✓	✓	✓	✓	✓	✓	✓	✓	✓	✓	✓	✓	✓	✓	✓	✓	✓
Ethyl 2-Methyl butanoate	108-64-5	MS, RI					✓	✓		✓				✓	✓	✓	✓	✓	✓	✓	✓	✓	✓
Ethyl 2-Methyl propanoate	97-62-1	MS, RI	✓	✓	✓	✓	✓	✓	✓	✓	✓	✓	✓	✓	✓	✓	✓	✓	✓	✓	✓	✓	✓
Ethyl 3-methylbutanoate	108-64-5	MS, RI		✓	✓	✓	✓	✓	✓	✓	✓		✓	✓	✓	✓	✓	✓	✓	✓	✓	✓	✓
Ethyl 4-oxopentanoate	539-88-8	MS, RI, ST		✓	✓	✓	✓	✓	✓	✓	✓	✓	✓	✓	✓	✓	✓	✓	✓	✓	✓	✓	✓
Ethyl acetate	141-78-6	MS, RI, ST	✓	✓	✓	✓	✓	✓	✓	✓	✓	✓	✓	✓	✓	✓	✓	✓	✓	✓	✓	✓	✓
Ethyl butanoate	105-54-4	MS, RI, ST	✓	✓	✓	✓	✓	✓	✓	✓	✓	✓	✓	✓	✓	✓	✓	✓	✓	✓	✓	✓	✓
Ethyl decanoate	110-38-3	MS, RI, ST	✓	✓	✓	✓	✓	✓	✓	✓	✓	✓	✓	✓	✓	✓	✓	✓	✓	✓	✓	✓	✓
Ethyl formate	109-94-4	MS, RI				✓	✓	✓	✓		✓	✓	✓	✓	✓	✓	✓	✓	✓	✓	✓	✓	✓
Ethyl hexadecanoate	628-97-7	MS, RI		✓	✓	✓	✓	✓	✓	✓	✓	✓	✓	✓	✓	✓	✓	✓	✓	✓	✓	✓	✓
Ethyl hexanoate	123-66-0	MS, RI, ST	✓	✓	✓	✓	✓	✓	✓	✓	✓	✓	✓	✓	✓	✓	✓	✓		✓	✓	✓	✓
Ethyl octanoate	106-32-1	MS, RI, ST		✓	✓	✓	✓	✓	✓	✓	✓	✓	✓	✓	✓	✓	✓	✓	✓	✓	✓	✓	✓
Ethyl pentadecanoate	41114-00-5	MS, RI	✓	✓	✓	✓	✓	✓	✓	✓	✓	✓	✓	✓	✓	✓	✓	✓	✓	✓	✓	✓	✓
Ethyl pentanoate	539-82-2	MS, RI		✓	✓	✓	✓	✓	✓	✓	✓	✓	✓	✓		✓	✓	✓	✓	✓	✓	✓	✓
Ethyl propanoate	105-37-3	MS, RI, ST		✓	✓	✓	✓	✓	✓	✓	✓	✓	✓	✓	✓	✓	✓	✓	✓	✓	✓	✓	✓
Ethyl tetradecanoate	124-06-1	MS, RI	✓	✓	✓	✓	✓	✓	✓	✓	✓	✓	✓	✓	✓	✓	✓	✓	✓	✓	✓	✓	✓
Methyl 2-hydroxybenzoate	119-36-8	MS, RI, ST		✓	✓	✓	✓	✓	✓	✓	✓	✓	✓	✓	✓	✓	✓	✓	✓	✓	✓	✓	✓
Methyl hexadecanoate	112-39-0	MS, RI	✓	✓	✓	✓	✓	✓	✓	✓										✓	✓	✓	
Methyl nonanoate	1731-84-6	MS, RI									✓	✓	✓							✓		✓	✓
Pentyl 2-hydroxypropanoate	6382-06-5	MS				✓		✓			✓	✓	✓	✓	✓	✓	✓	✓	✓	✓	✓	✓	✓
Prop-2-enyl 2-phenylacetate	1797-74-6	MS, RI				✓			✓		✓	✓	✓	✓	✓	✓	✓	✓	✓	✓	✓	✓	✓
**Phenols**																							
2,3,6-trimethylphenol	2416-94-6	MS, RI		✓	✓	✓	✓	✓	✓	✓	✓									✓	✓	✓	✓
2,6-dimethoxy-4-prop-2-enylphenol	6627-88-9	MS, RI				✓	✓	✓	✓	✓	✓	✓	✓	✓	✓	✓	✓	✓	✓	✓	✓	✓	✓
2,6-ditert-butyl-4-methylphenol	128-37-0	MS, RI		✓	✓	✓	✓	✓	✓	✓	✓		✓	✓	✓	✓	✓	✓	✓	✓	✓	✓	✓
2-methoxy-4-methylphenol	93-51-6	MS, RI		✓	✓	✓	✓	✓	✓	✓	✓	✓	✓	✓	✓	✓	✓	✓	✓	✓	✓	✓	✓
2,6-Dimethoxyphenol	91-10-1	MS, RI												✓	✓	✓	✓	✓	✓	✓	✓	✓	✓
2-methoxy-4-prop-2-enylphenol	97-53-0	MS, RI, ST		✓	✓	✓	✓	✓	✓	✓	✓	✓	✓	✓	✓	✓	✓	✓	✓	✓	✓	✓	✓
2-methoxy-4-propylphenol	2785-87-7	MS, RI				✓	✓	✓	✓	✓	✓	✓	✓		✓	✓	✓	✓	✓	✓	✓	✓	✓
2-methoxyphenol	90-05-1	MS, RI, ST			✓	✓	✓	✓	✓	✓	✓	✓	✓	✓	✓	✓	✓	✓	✓	✓	✓	✓	
2-methyl-5-propan-2-ylphenol	499-75-2	MS, RI, ST		✓	✓	✓	✓	✓	✓	✓	✓	✓	✓	✓	✓	✓	✓	✓	✓	✓	✓	✓	✓
2-*tert*-butylphenol	88-18-6	MS				✓	✓	✓	✓	✓	✓	✓	✓	✓	✓	✓	✓	✓	✓	✓	✓	✓	✓
4-ethyl-2-methoxyphenol	2785-89-9	MS, RI		✓	✓	✓	✓	✓	✓	✓	✓	✓	✓	✓	✓	✓	✓	✓	✓	✓	✓	✓	✓
4-prop-2-enylphenol	501-92-8	MS, RI				✓	✓	✓		✓				✓	✓	✓	✓	✓	✓	✓	✓	✓	✓
2-isopropyl-5-methylphenol	89-83-9	MS, RI		✓	✓	✓	✓	✓	✓	✓	✓	✓	✓	✓	✓	✓	✓	✓	✓	✓	✓	✓	✓
**Furans**																							
(5-formylfuran-2-yl)methyl acetate	10551-58-3	MS									✓	✓	✓	✓	✓	✓	✓	✓	✓	✓	✓	✓	✓
1-(2-furanyl) ethanone	1192-62-7	MS, RI, ST			✓	✓	✓	✓		✓	✓	✓	✓	✓	✓	✓	✓	✓	✓	✓	✓	✓	✓
2-butylfuran	4466-24-4	MS, RI	✓			✓	✓	✓	✓	✓	✓	✓	✓	✓						✓	✓	✓	✓
2-methyltetrahydrofuran-3-one	3188-00-9	MS, RI, ST	✓	✓	✓	✓	✓	✓	✓	✓	✓	✓	✓	✓	✓	✓	✓	✓	✓	✓	✓	✓	✓
5-(hydroxymethyl)furan-2-carboxaldehyde	67-47-0	MS, RI, ST				✓	✓	✓	✓				✓		✓	✓				✓	✓	✓	✓
5-methylfuran-2-carboxaldehyde	620-02-0	MS, RI, ST	✓	✓	✓	✓	✓	✓	✓	✓	✓	✓	✓	✓	✓	✓	✓	✓	✓	✓	✓	✓	✓
Furan-2-carboxaldehyde	98-01-1	MS, RI, ST	✓	✓	✓	✓	✓	✓	✓	✓	✓	✓	✓	✓	✓	✓	✓	✓	✓	✓	✓	✓	✓
Furan-2-yl methanol	98-00-0	MS, RI, ST				✓	✓						✓		✓	✓	✓	✓	✓	✓	✓	✓	✓
Furan-2-ylmethyl formate	13493-97-5	MS, RI				✓	✓	✓	✓		✓			✓	✓	✓	✓	✓	✓	✓	✓	✓	✓
**Terpenes**																							
1-methyl-4-propan-2-yl-7-oxabicyclo [2.2.1]heptane	470-67-7	MS, RI				✓	✓	✓	✓	✓	✓	✓								✓	✓	✓	✓
1-methyl-4-propan-2-ylbenzene	99-87-6	MS, RI		✓	✓	✓	✓	✓	✓	✓	✓	✓	✓	✓	✓	✓	✓	✓	✓	✓	✓	✓	✓
2-(4-methyl-1-cyclohex-3-enyl)propan-2-ol	98-55-5	MS, RI, ST		✓	✓	✓	✓	✓	✓	✓	✓	✓	✓	✓	✓	✓	✓	✓	✓	✓	✓	✓	✓
2-(5-ethenyl-5-methyloxolan-2-yl)propan-2-ol	5989-33-3	MS, RI		✓	✓	✓	✓	✓	✓	✓	✓	✓	✓	✓	✓	✓	✓	✓	✓	✓	✓	✓	✓
2-[(2R,5R)-5-methyl-5-vinyltetrahydrofuran-2-yl]propan-2-ol	68780-91-6	MS, RI		✓	✓	✓	✓	✓	✓	✓	✓	✓	✓	✓	✓	✓	✓	✓	✓	✓	✓	✓	✓
3,7-dimethylocta-1,6-dien-3-ol	78-70-6	MS, RI, ST	✓	✓	✓	✓	✓	✓	✓	✓	✓	✓	✓	✓	✓	✓	✓	✓	✓	✓	✓	✓	✓
1-methyl-4-propan-2-ylcyclohex-3-en-1-ol	586-82-3	MS, RI					✓	✓	✓					✓	✓	✓	✓	✓	✓	✓	✓	✓	✓
4-methyl-1-propan-2-ylcyclohex-3-en-1-ol	562-74-3	MS, RI, ST		✓	✓	✓	✓	✓	✓	✓	✓	✓	✓	✓	✓	✓	✓	✓	✓	✓	✓	✓	✓
2,6,6-trimethylcyclohexene-1-carbaldehyde	432-25-7	MS, RI	✓	✓	✓	✓	✓	✓	✓	✓	✓	✓	✓	✓	✓	✓	✓	✓	✓	✓	✓	✓	✓
3,3-dimethyl-2-methylidenebicyclo [2.2.1]heptane	79-92-5	MS, RI				✓	✓	✓	✓	✓	✓	✓	✓	✓		✓	✓	✓	✓	✓	✓	✓	✓
2-(4-methylcyclohex-3-en-1-yl)propan-2-ol	8000-41-7	MS, RI		✓	✓	✓	✓	✓	✓	✓	✓	✓	✓	✓	✓	✓	✓	✓	✓	✓	✓	✓	✓
(1S,8aR)-4,7-dimethyl-1-propan-2-yl-1,2,3,5,6,8a-hexahydronaphthalene	483-76-1	MS, RI			✓	✓	✓	✓		✓	✓									✓	✓	✓	✓
3,7-dimethyloct-6-en-1-ol	106-22-9	MS, RI	✓	✓	✓	✓	✓	✓	✓	✓	✓	✓	✓	✓	✓	✓	✓	✓	✓	✓	✓	✓	✓
(2Z)-3,7-dimethylocta-2,6-dien-1-ol	106-25-2	MS, RI		✓	✓	✓	✓	✓	✓	✓	✓	✓	✓	✓	✓	✓	✓	✓	✓	✓	✓	✓	✓
(2E)-3,7-dimethylocta-2,6-dien-1-ol	106-24-1	MS, RI	✓	✓	✓	✓	✓	✓	✓	✓	✓	✓	✓	✓	✓	✓	✓	✓	✓	✓	✓	✓	✓
3,7,11-trimethyl-1,6,10-dodecatrien-3-ol	142-50-7	MS, RI, ST	✓	✓	✓	✓	✓	✓	✓	✓	✓	✓	✓	✓	✓	✓	✓	✓	✓	✓	✓	✓	✓
2-[(2S,5S)-5-methyl-5-(4-methylcyclohex-3-en-1-yl)oxolan-2-yl]propan-2-ol; (3S,6S)-2,2,6-trimethyl-6-[(1S)-4-methylcyclohex-3-en-1-yl]oxan-3-ol	11087-43-7	MS		✓	✓	✓	✓	✓	✓	✓	✓			✓	✓	✓	✓		✓	✓	✓	✓	✓
(1R,4S,4aR,8aS)-4-Isopropyl-1,6-dimethyl-1,2,3,4,4a,7,8,8a-octahydro-1-naphthalenol	19435-97-3	MS, RI	✓	✓	✓	✓	✓	✓	✓	✓	✓				✓	✓	✓	✓	✓	✓	✓	✓	✓
(2E,6E)-3,7,11-trimethyldodeca-2,6,10-trien-1-ol	4602-84-0	MS, RI, ST	✓	✓	✓	✓	✓	✓	✓	✓	✓	✓	✓	✓	✓	✓	✓	✓	✓	✓	✓	✓	✓
**Divers**																							
[2S-[2alpha,5beta(E)]]-5-(1,5-Dimethyl-1,4-hexadienyl)-2-ethenyltetrahydro-2-methylfuran	72505-38-5	MS				✓	✓	✓	✓			✓	✓	✓	✓	✓	✓	✓	✓	✓	✓	✓	✓
4,4-dimethyl-8-methylene-1-oxaspiro [2.5]octane	54345-56-1	MS						✓		✓	✓			✓			✓			✓	✓	✓	✓
3-furanacetic acid, 4-hexyl-2,5-dihydro-2,5-dioxo	39212-21-0	MS		✓		✓	✓	✓	✓	✓										✓	✓	✓	✓
1-(2,6-dihydroxy-4-methoxyphenyl) ethanone	7507-89-3	MS				✓		✓				✓	✓	✓	✓	✓	✓	✓	✓	✓	✓	✓	✓
benzene-1,4-diol	123-31-9	MS, RI				✓				✓	✓									✓	✓	✓	✓
(3-hydroxyphenyl) acetate	102-29-4	MS		✓	✓	✓	✓	✓	✓	✓	✓	✓	✓	✓	✓	✓	✓	✓	✓	✓	✓	✓	✓
1,2,3,4,4a,5,6,7,8,8a-decahydronaphthalene	91-17-8	MS					✓			✓		✓								✓	✓		✓
1,1,6-trimethyl-2H-naphthalene	30364-38-6	MS, RI		✓		✓	✓	✓	✓	✓	✓				✓	✓		✓		✓	✓	✓	✓
1,6-dimethylnaphthalene	575-43-9	MS, RI		✓	✓	✓	✓	✓	✓	✓	✓		✓	✓			✓			✓	✓	✓	✓
3-methylpent-2-ene	922-61-2	MS, RI		✓	✓	✓	✓	✓	✓	✓	✓	✓	✓	✓	✓	✓	✓	✓	✓	✓	✓	✓	✓
2,2,6-trimethyl-6-vinyltetrahydro-2H-pyran	14049-11-7	MS, RI		✓		✓	✓	✓	✓	✓	✓			✓	✓	✓	✓	✓	✓	✓	✓	✓	✓
**Lactones**																							
1-(5-methylfuran-2-yl)ethanone	1193-79-9	MS, RI				✓	✓	✓		✓	✓	✓	✓	✓	✓	✓	✓	✓	✓	✓	✓	✓	✓
5-propyloxolan-2-one	105-21-5	MS, RI					✓		✓	✓	✓									✓	✓	✓	✓
5-butyl-4-methyloxolan-2-one	39212-23-2	MS, RI								✓	✓			✓	✓					✓	✓	✓	✓
5-methyl-3H-furan-2-one	591-12-8	MS, RI, ST	✓	✓	✓	✓	✓	✓	✓	✓	✓	✓	✓	✓	✓	✓	✓			✓	✓	✓	✓
5-methyl decahydro 3(2H)furanone		MS				✓	✓	✓			✓	✓	✓	✓	✓	✓	✓	✓	✓	✓	✓	✓	✓

a CAS: Chemical Abstract Service registration number.

b MS: Compounds identified by the EI mass spectra in the Wiley 138 library, RI: Compounds identified by the Retention Index from the literature, and ST: Compounds identified by comparison of their EI mass spectra and retention time with the corresponding pure standards.

c Region: I, Allier; II, Limousin; III, Tronçais; IV, Centre de la France.

**Table 2:** Relative concentration (Mean ± Standard Deviation) of volatile compounds according to the barrels origin region.

Compound	Region^a^			
	I	II	III	IV
**Acetals**				
1,1-diethoxy-ethane	1.540 ± 0.517	1.428 ± 0.553	1.541 ± 0.353	1.687 ± 0.688
1,1-diethoxyhexane	0.099 ± 0.058	0.098 ± 0.057	0.101 ± 0.055	0.110 ± 0.062
1,1-diethoxypropane	0.168 ± 0.054	0.185 ± 0.042	0.191 ± 0.055	0.183 ± 0.059
**Acids**				
3-methylbutanoic acid	0.114 ± 0.054	0.129 ± 0.038	0.127 ± 0.032	0.142 ± 0.041
Decanoic acid	1.762 ± 0.902	2.024 ± 0.790	2.018 ± 0.937	2.045 ± 0.876
Dodecanoic acid	0.478 ± 0.518	0.380 ± 0.264	0.369 ± 0.309	0.439 ± 0.303
Ethanoic acid	1.012 ± 0.507	1.067 ± 0.510	1.031 ± 0.439	1.056 ± 0.439
Hexadecanoic acid	0.087 ± 0.188	0.080 ± 0.149	0.042 ± 0.049	0.116 ± 0.205
Hexanoic acid	0.121 ± 0.055	0.117 ± 0.044	0.101 ± 0.031	0.117 ± 0.066
Octanoic acid	1.194 ± 0.622	1.525 ± 0.431	1.550 ± 0.518	1.558 ± 0.419
Propanoic acid	0.038 ± 0.023	0.030 ± 0.028	0.030 ± 0.027	0.086 ± 0.093
Tetradecanoic acid	0.051 ± 0.058	0.043 ± 0.042	0.049 ± 0.060	0.056 ± 0.065
**Alcohols**				
(4-propan-2-ylphenyl) methanol	0.155 ± 0.076	0.188 ± 0.055	0.186 ± 0.063	0.184 ± 0.069
1-butanol	0.036 ± 0.011	0.042 ± 0.008	0.040 ± 0.007	0.042 ± 0.007
1-decanol	0.068 ± 0.022	0.071 ± 0.022	0.073 ± 0.026	0.092 ± 0.036
1-dodecanol	0.032 ± 0.012	0.033 ± 0.009	0.035 ± 0.010	0.034 ± 0.012
1-hexadecanol	0.025 ± 0.020	0.025 ± 0.017	0.026 ± 0.023	0.027 ± 0.028
1-hexanol	0.362 ± 0.798	0.164 ± 0.053	0.179 ± 0.036	0.182 ± 0.022
1-propanol	0.359 ± 0.144	0.387 ± 0.119	0.373 ± 0.092	0.368 ± 0.114
1-tetradecanol	0.079 ± 0.044	0.089 ± 0.043	0.089 ± 0.055	0.084 ± 0.059
2-methyl propan-1-ol	3.321 ± 1.101	3.624 ± 0.637	3.618 ± 0.607	3.736 ± 0.307
2-phenylethanol	2.493 ± 1.192	2.845 ± 1.004	2.979 ± 0.788	3.001 ± 0.655
3-methylbutan-1-ol	43.97 ± 15.29	46.42 ± 8.15	46.26 ± 7.43	48.81 ± 5.86
3-octanol	0.039 ± 0.041	0.032 ± 0.013	0.035 ± 0.013	0.033 ± 0.011
3-phenylpropan-1-ol	0.266 ± 0.227	0.233 ± 0.090	0.234 ± 0.110	0.226 ± 0.096
Pent-4-en-1-ol	0.045 ± 0.056	0.032 ± 0.008	0.033 ± 0.008	0.032 ± 0.011
**Aldehydes**				
4-hydroxy-3,5-dimethoxybenzaldehyde	0.076 ± 0.092	0.047 ± 0.054	0.043 ± 0.022	0.060 ± 0.065
4-hydroxy-3-methoxybenzaldehyde	0.028 ± 0.014	0.032 ± 0.011	0.034 ± 0.012	0.031 ± 0.012
**Ketones**				
Cyclopentanone	0.062 ± 0.014	0.058 ± 0.023	0.067 ± 0.011	0.069 ± 0.017
**Esters**				
Acetic acid 2-phenylethyl ester	0.265 ± 0.095	0.292 ± 0.109	0.315 ± 0.102	0.318 ± 0.087
3-methylbutyl acetate	0.357 ± 0.140	0.406 ± 0.070	0.421 ± 0.072	0.438 ± 0.062
Butanedioic acid, diethyl Ester	0.089 ± 0.018	0.087 ± 0.028	0.095 ± 0.032	0.088 ± 0.023
Ethyl (2S)-2-hydroxypropanoate	2.883 ± 0.901	3.050 ± 0.843	3.171 ± 0.547	3.260 ± 0.328
Ethyl (9Z,12Z)-octadeca-9,12-dienoate	0.206 ± 0.318	0.351 ± 0.558	0.326 ± 0.644	0.369 ± 0.732
Ethyl (9Z,12Z,15Z)-octadeca-9,12,15-trienoate	0.042 ± 0.060	0.042 ± 0.058	0.039 ± 0.069	0.043 ± 0.072
Ethyl (Z)-octadec-9-enoate	0.060 ± 0.085	0.047 ± 0.074	0.043 ± 0.087	0.053 ± 0.104
Ethyl acetate	11.41 ± 2.52	11.86 ± 2.66	11.26 ± 2.02	12.00 ± 1.97
Ethyl butanoate	0.085 ± 0.024	0.087 ± 0.019	0.086 ± 0.017	0.095 ± 0.028
Ethyl decanoate	1.926 ± 0.933	2.186 ± 0.798	2.236 ± 0.946	1.971 ± 0.899
Ethyl dodecanoate	1.009 ± 0.460	1.006 ± 0.469	1.063 ± 0.547	0.853 ± 0.608
Ethyl hexadecanoate	0.662 ± 0.740	0.651 ± 0.708	0.674 ± 0.756	0.655 ± 0.900
Ethyl hexanoate	0.170 ± 0.054	0.163 ± 0.047	0.161 ± 0.034	0.177 ± 0.040
Ethyl octanoate	0.646 ± 0.244	0.718 ± 0.172	0.737 ± 0.203	0.731 ± 0.125
Ethyl propanoate	0.514 ± 0.202	0.580 ± 0.208	0.533 ± 0.143	0.570 ± 0.167
**Phenols**				
2,6-ditert-butyl-4-methylphenol	0.083 ± 0.032	0.090 ± 0.033	0.091 ± 0.037	0.092 ± 0.032
2-methoxy-4-methylphenol	0.058 ± 0.066	0.054 ± 0.024	0.040 ± 0.015	0.049 ± 0.008
2-methoxyphenol + (trans)-3,7-dimethyl-2,6-octadien-1-ol	0.099 ± 0.062	0.131 ± 0.074	0.125 ± 0.068	0.284 ± 0.245
2-methyl-5-propan-2-ylphenol	0.260 ± 0.119	0.300 ± 0.090	0.295 ± 0.101	0.305 ± 0.085
4-prop-2-enylphenol	0.022 ± 0.012	0.016 ± 0.007	0.014 ± 0.006	0.021 ± 0.009
**Furans**				
1-(2-furanyl)ethanone	0.118 ± 0.034	0.126 ± 0.023	0.133 ± 0.027	0.135 ± 0.028
2-methyltetrahydrofuran-3-one	0.091 ± 0.034	0.096 ± 0.021	0.095 ± 0.021	0.103 ± 0.019
5-methylfuran-2-carboxaldehyde	0.854 ± 0.411	0.850 ± 0.385	1.125 ± 0.234	0.932 ± 0.259
Furan-2-carboxaldehyde	2.483 ± 0.826	2.411 ± 0.672	2.781 ± 0.685	2.360 ± 0.596
**Terpenes**				
2-[(2R,5R)-5-methyl-5-vinyltetrahydrofuran-2-yl]propan-2-ol	0.255 ± 0.465	0.144 ± 0.037	0.145 ± 0.044	0.149 ± 0.038
3,7-dimethylocta-1,6-dien-3-ol	0.263 ± 0.073	0.282 ± 0.086	0.287 ± 0.100	0.378 ± 0.254
4-methyl-1-propan-2-ylcyclohex-3-en-1-ol	0.130 ± 0.042	0.139 ± 0.044	0.148 ± 0.038	0.143 ± 0.029
2-(4-methylcyclohex-3-en-1-yl)propan-2-ol	0.575 ± 0.194	0.650 ± 0.155	0.657 ± 0.182	0.652 ± 0.132
3,7-dimethyloct-6-en-1-ol	0.101 ± 0.032	0.113 ± 0.035	0.117 ± 0.040	0.067 ± 0.048
(2E,6E)-3,7,11-trimethyldodeca-2,6,10-trien-1-ol	0.087 ± 0.057	0.095 ± 0.053	0.096 ± 0.066	0.091 ± 0.073
**Divers**				
3-methylpent-2-ene	0.277 ± 0.086	0.308 ± 0.050	0.313 ± 0.060	0.300 ± 0.080
NI (184 PM)	0.262 ± 0.102	0.294 ± 0.083	0.299 ± 0.100	0.301 ± 0.077
**Lactones**				
5-methyl-3H-furan-2-one	0.234 ± 0.069	0.258 ± 0.057	0.259 ± 0.069	0.244 ± 0.085

a Region: I, Allier; II, Limousin; III, Tronçais; IV, Centre de la France.

**Table 3:** Relative concentration (Mean ± Standard Deviation) of volatile compounds according to maturation time in weeks.

Compound	Maturation time (weeks)																
	2	4	6	8	10	12		14	16	18	20	22	24	25	28	30	32
**Acetals**																	
1,1-diethoxy-ethane	1.139 ± 0.417	1.404 ± 0.206	1.873 ± 0.086	1.809 ± 0.098	2.037 ± 0.190	2.049 ± 0.184		1.879 ± 0.096	1.743 ± 0.254	1.056 ± 0.631	1.168 ± 0.764	1.671 ± 0.247	0.812 ± 0.713	1.446 ± 0.918	1.650 ± 0.165	1.550 ± 0.509	1.709 ± 0.540
1,1-diethoxyhexane	0.054 ± 0.014	0.062 ± 0.008	0.070 ± 0.002	0.072 ± 0.005	0.062 ± 0.006	0.065 ± 0.005		0.065 ± 0.005	0.064 ± 0.006	0.065 ± 0.014	0.058 ± 0.014	0.193 ± 0.016	0.196 ± 0.013	0.174 ± 0.051	0.132 ± 0.043	0.126 ± 0.055	0.175 ± 0.016
1,1-diethoxypropane	0.193 ± 0.063	0.215 ± 0.021	0.233 ± 0.004	0.186 ± 0.103	0.211 ± 0.007	0.209 ± 0.012		0.212 ± 0.004	0.215 ± 0.002	0.2160.016	0.226 ± 0.006	0.145 ± 0.007	0.145 ± 0.015	0.109 ± 0.061	0.140 ± 0.025	0.129 ± 0.036	0.138 ± 0.008
**Acids**																	
3-methylbutanoic acid	0.072 ± 0.056	0.094 ± 0.028	0.111 ± 0.001	0.101 ± 0.027	0.149 ± 0.011	0.159 ± 0.017		0.167 ± 0.024	0.165 ± 0.008	0.212 ± 0.018	0.158 ± 0.041	0.112 ± 0.005	0.111 ± 0.007	0.111 ± 0.009	0.105 ± 0.038	0.103 ± 0.029	0.111 ± 0.005
Decanoic acid	2.702 ± 1.016	3.283 ± 0.265	2.658 ± 0.063	2.035 ± 1.310	2.437 ± 0.218	2.206 ± 0.235		2.424 ± 0.044	2.618 ± 0.150	2.423 ± 0.254	2.585 ± 0.282	0.937 ± 0.129	1.213 ± 0.151	1.050 ± 0.110	1.112 ± 0.296	1.005 ± 0.308	1.028 ± 0.098
Dodecanoic acid	0.841 ± 0.310	1.427 ± 0.683	0.527 ± 0.010	0.513 ± 0.044	0.534 ± 0.069	0.364 ± 0.189		0.518 ± 0.010	0.583 ± 0.066	0.210 ± 0.286	0.207 ± 0.251	0.152 ± 0.032	0.258 ± 0.071	0.168 ± 0.084	0.196 ± 0.128	0.191 ± 0.064	0.192 ± 0.037
Ethanoic acid	0.186 ± 0.099	0.377 ± 0.047	0.768 ± 0.014	0.644 ± 0.298	0.735 ± 0.044	0.892 ± 0.179		0.806 ± 0.108	0.788 ± 0.048	1.271 ± 0.142	1.171 ± 0.033	1.564 ± 0.189	1.326 ± 0.187	1.520 ± 0.208	1.520 ± 0.298	1.385 ± 0.488	1.583 ± 0.124
Hexadecanoic acid	0.277 ± 0.213	0.663 ± 0.104	0.010 ± 0.001	0.011 ± 0.005	0.017 ± 0.022	0.012 ± 0.002		0.015 ± 0.004	0.021 ± 0.012	0.032 ± 0.007	0.060 ± 0.034	0.032 ± 0.008	0.066 ± 0.034	0.032 ± 0.025	0.117 ± 0.101	0.024 ± 0.009	0.037 ± 0.003
Hexanoic acid	0.075 ± 0.035	0.084 ± 0.014	0.142 ± 0.041	0.100 ± 0.010	0.099 ± 0.044	0.215 ± 0.074		0.096 ± 0.064	0.099 ± 0.053	0.113 ± 0.016	0.139 ± 0.046	0.167 ± 0.006	0.135 ± 0.043	0.115 ± 0.060	0.106 ± 0.039	0.076 ± 0.044	0.108 ± 0.055
Octanoic acid	1.520 ± 0.589	1.431 ± 0.808	1.980 ± 0.012	1.568 ± 0.900	1.753 ± 0.116	1.664 ± 0.154		1.783 ± 0.067	1.844 ± 0.071	1.864 ± 0.148	1.972 ± 0.189	0.996 ± 0.095	1.112 ± 0.085	0.787 ± 0.508	1.104 ± 0.182	0.972 ± 0.279	1.018 ± 0.052
Propanoic acid	0.000 ± 0.000	0.000 ± 0.000	0.031 ± 0.031	0.010 ± 0.008	0.086 ± 0.128	0.112 ± 0.164		0.039 ± 0.030	0.035 ± 0.035	0.019 ± 0.002	0.033 ± 0.027	0.058 ± 0.005	0.056 ± 0.005	0.061 ± 0.007	0.058 ± 0.021	0.095 ± 0.078	0.063 ± 0.006
Tetradecanoic acid	0.163 ± 0.071	0.214 ± 0.044	0.038 ± 0.002	0.031 ± 0.010	0.040 ± 0.011	0.032 ± 0.019		0.043 ± 0.007	0.045 ± 0.012	0.036 ± 0.022	0.058 ± 0.018	0.009 ± 0.003	0.030 ± 0.013	0.014 ± 0.003	0.034 ± 0.015	0.014 ± 0.006	0.016 ± 0.003
**Alcohols**																	
(4-propan-2-ylphenyl) methanol	0.200 ± 0.079	0.244 ± 0.023	0.234 ± 0.001	0.204 ± 0.072	0.234 ± 0.015	0.216 ± 0.020		0.229 ± 0.009	0.245 ± 0.009	0.202 ± 0.015	0.209 ± 0.017	0.123 ± 0.014	0.131 ± 0.010	0.094 ± 0.057	0.125 ± 0.024	0.091 ± 0.045	0.097 ± 0.052
1-butanol	0.029 ± 0.011	0.038 ± 0.005	0.046 ± 0.001	0.038 ± 0.016	0.040 ± 0.002	0.044 ± 0.003		0.038 ± 0.003	0.037 ± 0.002	0.049 ± 0.002	0.049 ± 0.007	0.044 ± 0.002	0.040 ± 0.004	0.035 ± 0.010	0.043 ± 0.007	0.036 ± 0.013	0.041 ± 0.004
1-decanol	0.072 ± 0.028	0.088 ± 0.010	0.114 ± 0.030	0.110 ± 0.028	0.084 ± 0.006	0.090 ± 0.018		0.093 ± 0.020	0.097 ± 0.017	0.096 ± 0.013	0.093 ± 0.006	0.048 ± 0.005	0.064 ± 0.025	0.044 ± 0.009	0.044 ± 0.009	0.046 ± 0.013	0.049 ± 0.005
1-dodecanol	0.040 ± 0.016	0.038 ± 0.011	0.041 ± 0.001	0.039 ± 0.001	0.042 ± 0.005	0.037 ± 0.004		0.038 ± 0.001	0.039 ± 0.003	0.037 ± 0.006	0.039 ± 0.006	0.031 ± 0.011	0.026 ± 0.008	0.029 ± 0.005	0.022 ± 0.009	0.023 ± 0.014	0.017 ± 0.006
1-hexadecanol	0.070 ± 0.032	0.079 ± 0.009	0.031 ± 0.003	0.029 ± 0.010	0.023 ± 0.007	0.020 ± 0.006		0.026 ± 0.002	0.028 ± 0.003	0.027 ± 0.001	0.031 ± 0.007	0.014 ± 0.014	0.009 ± 0.006	0.006 ± 0.001	0.011 ± 0.009	0.007 ± 0.002	0.007 ± 0.002
1-hexanol	0.136 ± 0.052	0.167 ± 0.018	0.211 ± 0.004	0.966 ± 1.517	0.182 ± 0.006	0.183 ± 0.006		0.185 ± 0.005	0.183 ± 0.003	0.210 ± 0.007	0.218 ± 0.002	0.109 ± 0.079	0.152 ± 0.016	0.158 ± 0.004	0.164 ± 0.012	0.141 ± 0.039	0.120 ± 0.076
1-propanol	0.219 ± 0.098	0.346 ± 0.017	0.394 ± 0.009	0.315 ± 0.174	0.323 ± 0.025	0.296 ± 0.037		0.293 ± 0.024	0.267 ± 0.023	0.463 ± 0.041	0.365 ± 0.016	0.499 ± 0.059	0.441 ± 0.052	0.474 ± 0.079	0.487 ± 0.137	0.389 ± 0.145	0.389 ± 0.193
1-tetradecanol	0.153 ± 0.060	0.180 ± 0.015	0.118 ± 0.003	0.110 ± 0.024	0.109 ± 0.016	0.090 ± 0.013		0.100 ± 0.006	0.113 ± 0.011	0.110 ± 0.015	0.106 ± 0.020	0.032 ± 0.006	0.041 ± 0.016	0.031 ± 0.008	0.029 ± 0.020	0.027 ± 0.012	0.036 ± 0.003
2-methyl propan-1-ol	2.559 ± 1.030	3.463 ± 0.228	4.287 ± 0.072	3.171 ± 2.093	3.710 ± 0.199	3.645 ± 0.224		3.613 ± 0.211	3.357 ± 0.178	3.713 ± 0.063	3.487 ± 0.163	3.971 ± 0.181	3.628 ± 0.286	3.798 ± 0.293	3.904 ± 0.678	3.303 ± 0.968	3.886 ± 0.151
2-phenylethanol	2.467 ± 0.904	3.054 ± 0.236	3.579 ± 0.023	2.664 ± 1.767	3.865 ± 0.151	3.608 ± 0.196		3.800 ± 0.104	3.937 ± 0.037	2.876 ± 0.182	2.966 ± 0.194	2.327 ± 0.123	2.377 ± 0.123	1.771 ± 1.084	2.366 ± 0.221	1.581 ± 1.115	2.314 ± 0.064
3-methylbutan-1-ol	30.67 ± 11.03	38.25 ± 2.88	45.60 ± 0.20	33.74 ± 22.48	45.04 ± 1.00	46.03 ± 0.30		44.96 ± 0.05	44.10 ± 0.51	47.82 ± 1.46	47.14 ± 0.51	54.87 ± 1.65	55.41 ± 2.66	54.02 ± 1.82	55.00 ± 2.13	46.50 ± 13.34	53.19 ± 0.38
3-octanol	0.027 ± 0.010	0.032 ± 0.007	0.051 ± 0.009	0.045 ± 0.019	0.042 ± 0.007	0.032 ± 0.001		0.041 ± 0.008	0.040 ± 0.007	0.033 ± 0.002	0.037 ± 0.002	0.023 ± 0.001	0.064 ± 0.081	0.023 ± 0.005	0.024 ± 0.004	0.020 ± 0.005	0.020 ± 0.005
3-phenylpropan-1-ol	0.310 ± 0.116	0.373 ± 0.023	0.298 ± 0.010	0.232 ± 0.145	0.281 ± 0.032	0.245 ± 0.031		0.267 ± 0.011	0.290 ± 0.028	0.289 ± 0.036	0.302 ± 0.042	0.118 ± 0.008	0.148 ± 0.028	0.344 ± 0.439	0.126 ± 0.046	0.112 ± 0.035	0.117 ± 0.013
Pent-4-en-1-ol	0.015 ± 0.011	0.029 ± 0.007	0.040 ± 0.000	0.091 ± 0.102	0.036 ± 0.004	0.040 ± 0.004		0.037 ± 0.003	0.038 ± 0.002	0.037 ± 0.002	0.040 ± 0.005	0.032 ± 0.002	0.020 ± 0.012	0.024 ± 0.009	0.027 ± 0.013	0.029 ± 0.007	0.031 ± 0.003
**Aldehydes**																	
4-hydroxy-3,5-dimethoxybenzaldehyde	0.166 ± 0.120	0.242 ± 0.016	0.027 ± 0.012	0.012 ± 0.004	0.046 ± 0.035	0.049 ± 0.012		0.068 ± 0.008	0.068 ± 0.018	0.053 ± 0.010	0.054 ± 0.003	0.015 ± 0.003	0.023 ± 0.018	0.015 ± 0.008	0.037 ± 0.021	0.029 ± 0.010	0.029 ± 0.005
4-hydroxy-3-methoxybenzaldehyde	0.022 ± 0.009	0.019 ± 0.013	0.021 ± 0.003	0.029 ± 0.003	0.037 ± 0.011	0.037 ± 0.016		0.048 ± 0.005	0.052 ± 0.006	0.039 ± 0.007	0.038 ± 0.003	0.019 ± 0.001	0.026 ± 0.005	0.020 ± 0.009	0.029 ± 0.005	0.027 ± 0.008	0.029 ± 0.007
**Ketones**																	
Cyclopentanone	0.042 ± 0.015	0.055 ± 0.008	0.075 ± 0.003	0.049 ± 0.030	0.071 ± 0.007	0.071 ± 0.002		0.068 ± 0.001	0.069 ± 0.003	0.073 ± 0.005	0.088 ± 0.026	0.068 ± 0.002	0.062 ± 0.005	0.060 ± 0.011	0.061 ± 0.011	0.063 ± 0.019	0.053 ± 0.030
**Esters**																	
Acetic acid 2-phenylethyl ester	0.310 ± 0.116	0.375 ± 0.032	0.415 ± 0.019	0.322 ± 0.197	0.371 ± 0.040	0.364 ± 0.035		0.359 ± 0.022	0.371 ± 0.028	0.324 ± 0.027	0.340 ± 0.026	0.221 ± 0.005	0.232 ± 0.020	0.215 ± 0.014	0.228 ± 0.049	0.143 ± 0.099	0.209 ± 0.012
3-methylbutyl acetate	0.272 ± 0.100	0.353 ± 0.022	0.428 ± 0.007	0.333 ± 0.183	0.416 ± 0.011	0.441 ± 0.018		0.457 ± 0.025	0.450 ± 0.014	0.502 ± 0.025	0.530 ± 0.057	0.392 ± 0.022	0.379 ± 0.021	0.343 ± 0.170	0.415 ± 0.055	0.372 ± 0.098	0.408 ± 0.023
Butanedioic acid, diethyl ester	0.061 ± 0.029	0.141 ± 0.004	0.121 ± 0.022	0.106 ± 0.009	0.092 ± 0.011	0.097 ± 0.008		0.103 ± 0.007	0.068 ± 0.006	0.084 ± 0.041	0.082 ± 0.002	0.087 ± 0.008	0.081 ± 0.009	0.100 ± 0.017	0.077 ± 0.023	0.083 ± 0.006	0.089 ± 0.018
Ethyl (2S)-2-hydroxypropanoate	2.033 ± 1.546	3.104 ± 0.270	3.768 ± 0.039	2.752 ± 1.809	3.346 ± 0.126	3.356 ± 0.121		3.357 ± 0.165	3.247 ± 0.098	3.441 ± 0.031	3.451 ± 0.056	3.118 ± 0.064	2.947 ± 0.155	3.040 ± 0.111	2.978 ± 0.233	2.728 ± 0.804	3.029 ± 0.082
Ethyl (9Z,12Z)-octadeca-9,12-dienoate	1.850 ± 0.650	1.409 ± 1.208	0.289 ± 0.044	0.260 ± 0.033	0.247 ± 0.042	0.122 ± 0.085		0.181 ± 0.044	0.198 ± 0.046	0.118 ± 0.080	0.149 ± 0.099	0.047 ± 0.005	0.094 ± 0.045	0.046 ± 0.025	0.081 ± 0.057	0.039 ± 0.028	0.047 ± 0.024
Ethyl (9Z,12Z,15Z)-octadeca-9,12,15-trienoate	0.196 ± 0.069	0.220 ± 0.013	0.031 ± 0.004	0.030 ± 0.012	0.031 ± 0.005	0.020 ± 0.009		0.026 ± 0.008	0.030 ± 0.006	0.016 ± 0.004	0.019 ± 0.010	0.019 ± 0.010	0.014 ± 0.009	0.008 ± 0.001	0.015 ± 0.006	0.009 ± 0.002	0.012 ± 0.008
Ethyl (Z)-octadec-9-enoate	0.256 ± 0.094	0.288 ± 0.004	0.031 ± 0.000	0.026 ± 0.012	0.029 ± 0.005	0.022 ± 0.006		0.023 ± 0.003	0.025 ± 0.003	0.017 ± 0.006	0.020 ± 0.007	0.009 ± 0.001	0.013 ± 0.006	0.053 ± 0.086	0.015 ± 0.007	0.010 ± 0.004	0.013 ± 0.007
Ethyl acetate	6.65 ± 2.18	8.21 ± 0.36	10.73 ± 0.51	10.65 ± 1.018	12.39 ± 1.12	12.55 ± 0.32		12.18 ± 0.66	12.54 ± 0.72	11.40 ± 0.91	11.01 ± 1.73	12.95 ± 0.74	12.75 ± 1.89	13.20 ± 0.75	12.20 ± 1.93	12.03 ± 3.55	14.47 ± 0.87
Ethyl butanoate	0.044 ± 0.017	0.061 ± 0.003	0.077 ± 0.001	0.070 ± 0.018	0.084 ± 0.005	0.086 ± 0.004		0.090 ± 0.007	0.090 ± 0.004	0.106 ± 0.007	0.113 ± 0.020	0.097 ± 0.006	0.110 ± 0.019	0.105 ± 0.008	0.105 ± 0.021	0.086 ± 0.024	0.083 ± 0.029
Ethyl decanoate	2.571 ± 0.940	3.143 ± 0.087	2.948 ± 0.109	1.532 ± 1.723	2.795 ± 0.254	2.507 ± 0.240		2.809 ± 0.089	2.943 ± 0.146	2.365 ± 0.264	2.490 ± 0.228	1.255 ± 0.031	1.347 ± 0.116	1.228 ± 0.107	1.269 ± 0.278	1.132 ± 0.329	1.242 ± 0.096
Ethyl dodecanoate	1.530 ± 0.549	1.830 ± 0.027	1.379 ± 0.059	1.358 ± 0.153	1.313 ± 0.169	0.792 ± 0.666		1.253 ± 0.069	1.349 ± 0.129	1.080 ± 0.209	1.246 ± 0.287	0.427 ± 0.039	0.526 ± 0.101	0.393 ± 0.261	0.354 ± 0.243	0.468 ± 0.141	0.483 ± 0.090
Ethyl hexadecanoate	2.353 ± 0.732	2.785 ± 0.027	0.700 ± 0.031	0.417 ± 0.421	0.763 ± 0.139	0.576 ± 0.102		0.648 ± 0.067	0.706 ± 0.096	0.420 ± 0.279	0.676 ± 0.171	0.135 ± 0.036	0.233 ± 0.088	0.118 ± 0.070	0.160 ± 0.071	0.125 ± 0.058	0.130 ± 0.049
Ethyl hexanoate	0.106 ± 0.035	0.122 ± 0.014	0.154 ± 0.002	0.154 ± 0.028	0.148 ± 0.005	0.152 ± 0.000		0.157 ± 0.007	0.156 ± 0.005	0.133 ± 0.005	0.153 ± 0.009	0.249 ± 0.040	0.218 ± 0.027	0.217 ± 0.037	0.193 ± 0.051	0.168 ± 0.054	0.208 ± 0.020
Ethyl octanoate	0.621 ± 0.232	0.791 ± 0.031	0.864 ± 0.031	0.665 ± 0.424	0.836 ± 0.050	0.790 ± 0.045		0.866 ± 0.005	0.881 ± 0.020	0.849 ± 0.072	0.897 ± 0.044	0.562 ± 0.010	0.571 ± 0.014	0.557 ± 0.037	0.566 ± 0.113	0.498 ± 0.138	0.552 ± 0.035
Ethyl propanoate	0.257 ± 0.089	0.389 ± 0.007	0.427 ± 0.017	0.334 ± 0.192	0.463 ± 0.016	0.499 ± 0.032		0.504 ± 0.026	0.492 ± 0.030	0.610 ± 0.022	0.588 ± 0.079	0.778 ± 0.112	0.705 ± 0.142	0.721 ± 0.098	0.675 ± 0.209	0.594 ± 0.232	0.746 ± 0.109
**Phenols**																	
2,6-ditert-butyl-4-methylphenol	0.098 ± 0.036	0.123 ± 0.007	0.125 ± 0.003	0.105 ± 0.042	0.118 ± 0.007	0.113 ± 0.007		0.120 ± 0.003	0.124 ± 0.005	0.095 ± 0.005	0.097 ± 0.003	0.053 ± 0.001	0.056 ± 0.005	0.052 ± 0.004	0.058 ± 0.009	0.047 ± 0.013	0.051 ± 0.006
2-methoxy-4-methylphenol	0.033 ± 0.015	0.040 ± 0.010	0.049 ± 0.001	0.067 ± 0.028	0.054 ± 0.007	0.060 ± 0.006		0.059 ± 0.002	0.100 ± 0.124	0.047 ± 0.009	0.051 ± 0.004	0.052 ± 0.035	0.040 ± 0.009	0.034 ± 0.019	0.060 ± 0.037	0.033 ± 0.011	0.035 ± 0.005
2-methoxyphenol. + (trans)-3,7-dimethyl-2,6-octadien-1-ol	0.073 ± 0.028	0.089 ± 0.010	0.187 ± 0.158	0.093 ± 0.012	0.211 ± 0.084	0.440 ± 0.455		0.180 ± 0.101	0.176 ± 0.082	0.254 ± 0.038	0.184 ± 0.110	0.081 ± 0.003	0.107 ± 0.043	0.168 ± 0.175	0.249 ± 0.301	0.071 ± 0.021	0.076 ± 0.001
2-methyl-5-propan-2-ylphenol	0.292 ± 0.111	0.420 ± 0.034	0.370 ± 0.006	0.307 ± 0.140	0.380 ± 0.028	0.343 ± 0.035		0.368 ± 0.009	0.399 ± 0.015	0.317 ± 0.028	0.330 ± 0.031	0.197 ± 0.012	0.222 ± 0.020	0.158 ± 0.089	0.206 ± 0.045	0.185 ± 0.058	0.193 ± 0.010
4-prop-2-enylphenol	0.022 ± 0.010	0.024 ± 0.006	0.023 ± 0.006	0.029 ± 0.016	0.022 ± 0.007	0.023 ± 0.008		0.022 ± 0.008	0.020 ± 0.005	0.014 ± 0.003	0.016 ± 0.003	0.016 ± 0.009	0.019 ± 0.019	0.014 ± 0.008	0.015 ± 0.006	0.010 ± 0.004	0.010 ± 0.002
**Furans**																	
1-(2-furanyl)ethanone	0.089 ± 0.031	0.113 ± 0.008	0.142 ± 0.009	0.111 ± 0.056	0.150 ± 0.011	0.149 ± 0.004		0.153 ± 0.005	0.153 ± 0.008	0.153 ± 0.026	0.153 ± 0.024	0.118 ± 0.002	0.111 ± 0.007	0.117 ± 0.012	0.116 ± 0.011	0.105 ± 0.030	0.116 ± 0.006
2-methyltetrahydrofuran-3-one	0.051 ± 0.019	0.066 ± 0.006	0.089 ± 0.002	0.091 ± 0.042	0.111 ± 0.022	0.098 ± 0.043		0.105 ± 0.018	0.096 ± 0.015	0.100 ± 0.004	0.102 ± 0.007	0.119 ± 0.029	0.108 ± 0.015	0.112 ± 0.015	0.105 ± 0.013	0.090 ± 0.026	0.098 ± 0.004
5-methylfuran-2-carboxaldehyde	0.506 ± 0.196	0.696 ± 0.077	0.946 ± 0.078	0.925 ± 0.109	1.109 ± 0.105	1.211 ± 0.098		1.221 ± 0.083	1.224 ± 0.095	1.210 ± 0.084	1.214 ± 0.081	0.328 ± 0.510	0.636 ± 0.490	0.996 ± 0.128	0.999 ± 0.163	0.539 ± 0.424	1.024 ± 0.099
Furan-2-carboxaldehyde	0.908 ± 0.371	1.530 ± 0.240	2.072 ± 0.200	1.876 ± 0.710	2.810 ± 0.282	3.328 ± 0.449		3.217 ± 0.248	3.141 ± 0.295	2.807 ± 0.167	2.780 ± 0.190	2.561 ± 0.182	2.466 ± 0.418	2.668 ± 0.373	2.532 ± 0.526	2.385 ± 0.575	2.727 ± 0.277
**Terpenes**																	
2-[(2R,5R)-5-methyl-5-vinyltetrahydrofuran-2-yl]propan-2-ol	0.148 ± 0.053	0.185 ± 0.013	0.192 ± 0.010	0.626 ± 0.871	0.166 ± 0.009	0.156 ± 0.006		0.166 ± 0.003	0.172 ± 0.002	0.159 ± 0.008	0.166 ± 0.003	0.102 ± 0.004	0.106 ± 0.005	0.103 ± 0.006	0.107 ± 0.014	0.095 ± 0.027	0.106 ± 0.011
3,7-dimethylocta-1,6-dien-3-ol	0.340 ± 0.229	0.344 ± 0.032	0.391 ± 0.009	0.356 ± 0.069	0.335 ± 0.018	0.322 ± 0.022		0.333 ± 0.004	0.342 ± 0.008	0.335 ± 0.024	0.341 ± 0.014	0.143 ± 0.095	0.367 ± 0.312	0.192 ± 0.016	0.204 ± 0.031	0.372 ± 0.416	0.146 ± 0.078
4-methyl-1-propan-2-ylcyclohex-3-en-1-ol	0.118 ± 0.082	0.166 ± 0.012	0.182 ± 0.005	0.143 ± 0.080	0.166 ± 0.012	0.157 ± 0.012		0.165 ± 0.004	0.171 ± 0.008	0.156 ± 0.015	0.160 ± 0.012	0.115 ± 0.004	0.118 ± 0.012	0.110 ± 0.012	0.116 ± 0.021	0.102 ± 0.029	0.110 ± 0.008
2-(4-methylcyclohex-3-en-1-yl)propan-2-ol	0.613 ± 0.226	0.748 ± 0.065	0.807 ± 0.023	0.631 ± 0.370	0.753 ± 0.049	0.707 ± 0.060		0.749 ± 0.009	0.775 ± 0.026	0.726 ± 0.059	0.750 ± 0.050	0.500 ± 0.015	0.515 ± 0.036	0.484 ± 0.036	0.503 ± 0.079	0.446 ± 0.126	0.480 ± 0.021
3,7-dimethyloct-6-en-1-ol	0.098 ± 0.029	0.123 ± 0.034	0.120 ± 0.083	0.107 ± 0.072	0.134 ± 0.008	0.089 ± 0.064		0.104 ± 0.057	0.114 ± 0.054	0.106 ± 0.066	0.136 ± 0.015	0.078 ± 0.003	0.071 ± 0.035	0.071 ± 0.015	0.083 ± 0.015	0.071 ± 0.021	0.076 ± 0.006
(2E,6E)-3,7,11-trimethyldodeca-2,6,10-trien-1-ol	0.196 ± 0.074	0.226 ± 0.014	0.126 ± 0.001	0.107 ± 0.052	0.118 ± 0.017	0.093 ± 0.021		0.103 ± 0.008	0.115 ± 0.013	0.106 ± 0.015	0.110 ± 0.018	0.026 ± 0.011	0.044 ± 0.023	0.031 ± 0.007	0.032 ± 0.017	0.032 ± 0.012	0.035 ± 0.006
**Divers**																	
3-methylpent-2-ene	0.250 ± 0.100	0.317 ± 0.032	0.368 ± 0.010	0.274 ± 0.171	0.322 ± 0.021	0.299 ± 0.015		0.247 ± 0.149	0.329 ± 0.013	0.342 ± 0.019	0.354 ± 0.011	0.302 ± 0.006	0.297 ± 0.014	0.290 ± 0.021	0.279 ± 0.043	0.249 ± 0.077	0.289 ± 0.009
NI (184 PM)	0.280 ± 0.106	0.344 ± 0.030	0.375 ± 0.007	0.288 ± 0.177	0.353 ± 0.021	0.325 ± 0.014		0.355 ± 0.010	0.371 ± 0.010	0.362 ± 0.025	0.374 ± 0.023	0.207 ± 0.010	0.223 ± 0.026	0.192 ± 0.022	0.206 ± 0.039	0.188 ± 0.055	0.203 ± 0.010
**Lactones**																	
5-methyl-3H-furan-2-one	0.220 ± 0.083	0.271 ± 0.031	0.317 ± 0.005	0.251 ± 0.122	0.234 ± 0.104	0.218 ± 0.122		0.293 ± 0.022	0.298 ± 0.011	0.334 ± 0.010	0.329 ± 0.006	0.211 ± 0.004	0.209 ± 0.011	0.203 ± 0.010	0.214 ± 0.015	0.180 ± 0.053	0.203 ± 0.009

**Table 4:** General Discriminant Analysis Classification table according to barrel regions.

Real Region^a^	Percent of correct classification	Predicted Region^a^			
		I	II	III	IV
I	86.7	13	0	2	0
II	81.3	3	13	0	0
III	92.3	1	0	12	0
IV	68.8	2	3	0	11
Total	81.7	19	16	14	11

a Region: I, Allier; II, Limousin; III, Tronçais; IV, Centre de la France.

**Table 5:** Tests of Significance of Squared Mahalanobis Distances (F tests with 8 and 49° of freedom) according to barrel origins. Sigma-restricted parameterization.

Region^a^	Region^a^					
	I		II		III	
	F	p	F	p	F	p
II	4.518	0.000				
III	5.536	0.000	15.625	0.000		
IV	8.524	0.000	7.111	0.000	13.970	0.000

a Region: I, Allier; II, Limousin; III, Tronçais; IV, Centre de la France.

**Table 6:** General discriminant analysis statistics for each tequila sample according to barrel origin (n = 60).

Sample^b^	Real Region^a^	Region of Classification	Probability of classification according to Region^a^			
			I	II	III	IV
I,2	I	I	0.879	0.108	0.012	0.002
I,4	I	I	0.682	0.061	0.009	0.248
I,4	I	I	0.987	0.011	0.000	0.001
*I,5	I	III	0.370	0.114	0.512	0.004
I,6	I	I	0.914	0.066	0.019	0.000
I,7	I	I	0.937	0.053	0.010	0.000
I,16	I	I	0.869	0.104	0.026	0.001
I,18	I	I	0.446	0.424	0.022	0.107
I,20	I	I	0.731	0.111	0.003	0.155
I,22	I	I	0.624	0.289	0.031	0.055
I,24	I	I	0.517	0.459	0.001	0.024
I,26	I	I	0.966	0.005	0.029	0.000
*I,28	I	III	0.327	0.001	0.670	0.002
I,30	I	I	0.855	0.011	0.133	0.002
I,32	I	I	0.920	0.032	0.045	0.003
*II,2	II	I	0.687	0.307	0.001	0.004
II,4	II	II	0.057	0.820	0.000	0.123
II,6	II	II	0.024	0.832	0.000	0.144
II,8	II	II	0.001	0.998	0.000	0.001
II,10	II	II	0.014	0.985	0.000	0.001
II,12	II	II	0.136	0.862	0.000	0.002
II,14	II	II	0.018	0.979	0.000	0.004
II,16	II	II	0.118	0.877	0.000	0.004
II,18	II	II	0.010	0.983	0.000	0.007
II,20	II	II	0.154	0.721	0.001	0.124
*II,22	II	I	0.775	0.184	0.018	0.023
II,24	II	II	0.004	0.972	0.000	0.024
II,26	II	II	0.236	0.748	0.001	0.014
II,28	II	II	0.000	0.993	0.000	0.006
*II,30	II	I	0.921	0.033	0.042	0.003
II,32	II	II	0.103	0.813	0.000	0.083
III,2	III	III	0.061	0.000	0.925	0.013
III,6	III	III	0.069	0.004	0.918	0.009
III,8	III	III	0.029	0.000	0.970	0.001
III,10	III	III	0.018	0.000	0.981	0.000
III,14	III	III	0.000	0.000	1.000	0.000
III,16	III	III	0.004	0.000	0.996	0.000
III,18	III	III	0.001	0.000	0.999	0.000
III,20	III	III	0.001	0.000	0.999	0.000
III,24	III	III	0.331	0.002	0.665	0.002
III,26	III	III	0.014	0.000	0.986	0.000
III,28	III	III	0.003	0.000	0.997	0.000
*III,30	III	I	0.963	0.006	0.030	0.000
III,32	III	III	0.432	0.003	0.563	0.001
*IV,2	IV	I	0.520	0.215	0.072	0.194
*IV,4	IV	II	0.249	0.379	0.000	0.371
IV,6	IV	IV	0.000	0.000	0.000	1.000
IV,4	IV	IV	0.001	0.001	0.000	0.998
IV,10	IV	IV	0.226	0.154	0.296	0.325
IV,12	IV	IV	0.000	0.000	0.000	1.000
IV,14	IV	IV	0.046	0.005	0.002	0.947
IV,16	IV	IV	0.014	0.007	0.002	0.976
IV,18	IV	IV	0.008	0.007	0.000	0.985
*IV,20	IV	II	0.017	0.577	0.000	0.406
*IV,22	IV	II	0.007	0.727	0.000	0.266
IV,24	IV	IV	0.000	0.000	0.000	1.000
IV,26	IV	IV	0.004	0.038	0.000	0.958
IV,28	IV	IV	0.000	0.000	0.000	1.000
IV,30	IV	IV	0.004	0.219	0.000	0.777
*IV,32	IV	I	0.482	0.257	0.015	0.246

a Region: I, Allier; II, Limousin; III, Tronçais; IV, Centre de la France.

b Sample expressed as R,W: Region, maturation time in weeks, samples marked with * were misclassified.

**Table 7:** General Discriminant Analysis Classification table according to Tequilas maturation time (weeks).

Real Maturation time (weeks)	Percent of correct classification	Maturation time (weeks)															
		2	4	6	8	10	12	14	16	18	20	22	24	26	28	30	32
2	100.0	4	0	0	0	0	0	0	0	0	0	0	0	0	0	0	0
4	100.0	0	3	0	0	0	0	0	0	0	0	0	0	0	0	0	0
6	100.0	0	0	3	0	0	0	0	0	0	0	0	0	0	0	0	0
8	75.0	0	0	1	3	0	0	0	0	0	0	0	0	0	0	0	0
10	100.0	0	0	0	0	4	0	0	0	0	0	0	0	0	0	0	0
12	100.0	0	0	0	0	0	3	0	0	0	0	0	0	0	0	0	0
14	75.0	0	0	0	0	0	1	3	0	0	0	0	0	0	0	0	0
16	100.0	0	0	0	0	0	0	0	4	0	0	0	0	0	0	0	0
18	100.0	0	0	0	0	0	0	0	0	4	0	0	0	0	0	0	0
20	100.0	0	0	0	0	0	0	0	0	0	4	0	0	0	0	0	0
22	100.0	0	0	0	0	0	0	0	0	0	0	3	0	0	0	0	0
24	100.0	0	0	0	0	0	0	0	0	0	0	0	4	0	0	0	0
26	100.0	0	0	0	0	0	0	0	0	0	0	0	0	4	0	0	0
28	100.0	0	0	0	0	0	0	0	0	0	0	0	0	0	4	0	0
30	100.0	0	0	0	0	0	0	0	0	0	0	0	0	0	0	4	0
32	100.0	0	0	0	0	0	0	0	0	0	0	0	0	0	0	0	4
Total	96.7	4	3	4	3	4	4	3	4	4	4	3	4	4	4	4	4

**Table 8:** Tests of Significance of Squared Mahalanobis Distances (F tests with 24 and 21° of freedom) according to the maturation time (weeks). Sigma-restricted parameterization.

Maturation time (weeks)	Maturation time (weeks)																													
	2		4		6		8		10		12		14		16		18		20		22		24		26		28		30	
	F	p	F	p	F	p	F	p	F	p	F	p	F	p	F	p	F	p	F	p	F	p	F	p	F	p	F	p	F	p
4	17.53	0.000																												
6	273.64	0.000	198.09	0.000																										
8	309.11	0.000	218.57	0.000	0.32	0.996																								
10	331.28	0.000	217.48	0.000	13.38	0.000	15.34	0.000																						
12	268.99	0.000	178.61	0.000	14.37	0.000	15.66	0.000	1.26	0.297																				
14	321.35	0.000	211.93	0.000	13.01	0.000	14.78	0.000	1.12	0.403	1.08	0.433																		
16	333.82	0.000	216.96	0.000	18.47	0.000	21.01	0.000	1.41	0.214	1.08	0.429	0.96	0.539																
18	353.74	0.000	245.71	0.000	38.03	0.000	45.66	0.000	36.10	0.000	35.63	0.000	36.98	0.000	40.20	0.000														
20	349.61	0.000	245.99	0.000	38.16	0.000	46.07	0.000	43.44	0.000	42.94	0.000	43.63	0.000	48.42	0.000	1.90	0.070												
22	140.98	0.000	87.35	0.000	84.26	0.000	92.27	0.000	77.56	0.000	60.26	0.000	78.94	0.000	80.53	0.000	79.42	0.000	86.81	0.000										
24	218.75	0.000	133.76	0.000	105.73	0.000	119.98	0.000	89.48	0.000	69.74	0.000	92.51	0.000	91.31	0.000	75.95	0.000	86.44	0.000	7.76	0.000								
26	222.05	0.000	135.85	0.000	105.70	0.000	120.00	0.000	88.85	0.000	69.03	0.000	91.65	0.000	90.45	0.000	75.55	0.000	86.23	0.000	7.54	0.000	1.02	0.486						
28	188.75	0.000	115.41	0.000	94.61	0.000	106.51	0.000	82.16	0.000	61.75	0.000	83.16	0.000	83.70	0.000	81.72	0.000	91.72	0.000	2.73	0.012	4.73	0.000	4.59	0.000				
30	199.05	0.000	122.84	0.000	89.97	0.000	101.32	0.000	77.11	0.000	59.05	0.000	79.22	0.000	79.69	0.000	70.11	0.000	79.83	0.000	4.48	0.000	3.25	0.004	3.57	0.002	2.42	0.022		
32	174.78	0.000	106.01	0.000	101.32	0.000	113.93	0.000	91.89	0.000	70.21	0.000	93.20	0.000	94.35	0.000	86.98	0.000	96.14	0.000	1.32	0.263	4.38	0.001	4.48	0.000	1.30	0.274	2.53	0.018

**Table 9:** General discriminant analysis statistics for each tequila sample according to the maturation time (n = 60).

Sample^a^	Real maturation time (weeks)	Maturation time (weeks) Classification	Probability of classification according to maturation time (weeks)															
			2	4	6	8	10	12	14	16	18	20	22	24	26	28	30	32
I,2	2	2	1.00	0.00	0.00	0.00	0.00	0.00	0.00	0.00	0.00	0.00	0.00	0.00	0.00	0.00	0.00	0.00
I,4	4	4	0.00	1.00	0.00	0.00	0.00	0.00	0.00	0.00	0.00	0.00	0.00	0.00	0.00	0.00	0.00	0.00
I,4	8	8	0.00	0.00	0.00	1.00	0.00	0.00	0.00	0.00	0.00	0.00	0.00	0.00	0.00	0.00	0.00	0.00
I,5	10	10	0.00	0.00	0.00	0.00	1.00	0.00	0.00	0.00	0.00	0.00	0.00	0.00	0.00	0.00	0.00	0.00
I,6	12	12	0.00	0.00	0.00	0.00	0.00	1.00	0.00	0.00	0.00	0.00	0.00	0.00	0.00	0.00	0.00	0.00
I,7	14	14	0.00	0.00	0.00	0.00	0.00	0.00	1.00	0.00	0.00	0.00	0.00	0.00	0.00	0.00	0.00	0.00
I,16	16	16	0.00	0.00	0.00	0.00	0.00	0.00	0.00	1.00	0.00	0.00	0.00	0.00	0.00	0.00	0.00	0.00
I,18	18	18	0.00	0.00	0.00	0.00	0.00	0.00	0.00	0.00	1.00	0.00	0.00	0.00	0.00	0.00	0.00	0.00
I,20	20	20	0.00	0.00	0.00	0.00	0.00	0.00	0.00	0.00	0.00	1.00	0.00	0.00	0.00	0.00	0.00	0.00
I,22	22	22	0.00	0.00	0.00	0.00	0.00	0.00	0.00	0.00	0.00	0.00	1.00	0.00	0.00	0.00	0.00	0.00
I,24	24	24	0.00	0.00	0.00	0.00	0.00	0.00	0.00	0.00	0.00	0.00	0.00	1.00	0.00	0.00	0.00	0.00
I,26	26	26	0.00	0.00	0.00	0.00	0.00	0.00	0.00	0.00	0.00	0.00	0.00	0.00	1.00	0.00	0.00	0.00
I,28	28	28	0.00	0.00	0.00	0.00	0.00	0.00	0.00	0.00	0.00	0.00	0.00	0.00	0.00	1.00	0.00	0.00
I,30	30	30	0.00	0.00	0.00	0.00	0.00	0.00	0.00	0.00	0.00	0.00	0.00	0.00	0.00	0.00	0.99	0.00
I,32	32	32	0.00	0.00	0.00	0.00	0.00	0.00	0.00	0.00	0.00	0.00	0.00	0.00	0.00	0.00	0.00	1.00
II,2	2	2	1.00	0.00	0.00	0.00	0.00	0.00	0.00	0.00	0.00	0.00	0.00	0.00	0.00	0.00	0.00	0.00
II,4	4	4	0.00	1.00	0.00	0.00	0.00	0.00	0.00	0.00	0.00	0.00	0.00	0.00	0.00	0.00	0.00	0.00
II,6	6	6	0.00	0.00	1.00	0.00	0.00	0.00	0.00	0.00	0.00	0.00	0.00	0.00	0.00	0.00	0.00	0.00
*II,8	8	6	0.00	0.00	0.71	0.29	0.00	0.00	0.00	0.00	0.00	0.00	0.00	0.00	0.00	0.00	0.00	0.00
II,10	10	10	0.00	0.00	0.00	0.00	1.00	0.00	0.00	0.00	0.00	0.00	0.00	0.00	0.00	0.00	0.00	0.00
II,12	12	12	0.00	0.00	0.00	0.00	0.00	1.00	0.00	0.00	0.00	0.00	0.00	0.00	0.00	0.00	0.00	0.00
*II,14	14	12	0.00	0.00	0.00	0.00	0.01	0.44	0.11	0.44	0.00	0.00	0.00	0.00	0.00	0.00	0.00	0.00
II,16	16	16	0.00	0.00	0.00	0.00	0.00	0.02	0.28	0.71	0.00	0.00	0.00	0.00	0.00	0.00	0.00	0.00
II,18	18	18	0.00	0.00	0.00	0.00	0.00	0.00	0.00	0.00	1.00	0.00	0.00	0.00	0.00	0.00	0.00	0.00
II,20	20	20	0.00	0.00	0.00	0.00	0.00	0.00	0.00	0.00	0.00	1.00	0.00	0.00	0.00	0.00	0.00	0.00
II,22	22	22	0.00	0.00	0.00	0.00	0.00	0.00	0.00	0.00	0.00	0.00	1.00	0.00	0.00	0.00	0.00	0.00
II,24	24	24	0.00	0.00	0.00	0.00	0.00	0.00	0.00	0.00	0.00	0.00	0.00	1.00	0.00	0.00	0.00	0.00
II,26	26	26	0.00	0.00	0.00	0.00	0.00	0.00	0.00	0.00	0.00	0.00	0.00	0.19	0.81	0.00	0.00	0.00
II,28	28	28	0.00	0.00	0.00	0.00	0.00	0.00	0.00	0.00	0.00	0.00	0.00	0.00	0.00	1.00	0.00	0.00
II,30	30	30	0.00	0.00	0.00	0.00	0.00	0.00	0.00	0.00	0.00	0.00	0.00	0.00	0.00	0.00	1.00	0.00
II,32	32	32	0.00	0.00	0.00	0.00	0.00	0.00	0.00	0.00	0.00	0.00	0.00	0.00	0.00	0.00	0.00	1.00
III,2	2	2	1.00	0.00	0.00	0.00	0.00	0.00	0.00	0.00	0.00	0.00	0.00	0.00	0.00	0.00	0.00	0.00
III,6	6	6	0.00	0.00	0.98	0.02	0.00	0.00	0.00	0.00	0.00	0.00	0.00	0.00	0.00	0.00	0.00	0.00
III,8	8	8	0.00	0.00	0.16	0.84	0.00	0.00	0.00	0.00	0.00	0.00	0.00	0.00	0.00	0.00	0.00	0.00
III,10	10	10	0.00	0.00	0.00	0.00	1.00	0.00	0.00	0.00	0.00	0.00	0.00	0.00	0.00	0.00	0.00	0.00
III,14	14	14	0.00	0.00	0.00	0.00	0.00	0.00	1.00	0.00	0.00	0.00	0.00	0.00	0.00	0.00	0.00	0.00
III,16	16	16	0.00	0.00	0.00	0.00	0.00	0.00	0.00	1.00	0.00	0.00	0.00	0.00	0.00	0.00	0.00	0.00
III,18	18	18	0.00	0.00	0.00	0.00	0.00	0.00	0.00	0.00	1.00	0.00	0.00	0.00	0.00	0.00	0.00	0.00
III,20	20	20	0.00	0.00	0.00	0.00	0.00	0.00	0.00	0.00	0.00	1.00	0.00	0.00	0.00	0.00	0.00	0.00
III,24	24	24	0.00	0.00	0.00	0.00	0.00	0.00	0.00	0.00	0.00	0.00	0.00	0.80	0.20	0.00	0.00	0.00
III,26	26	26	0.00	0.00	0.00	0.00	0.00	0.00	0.00	0.00	0.00	0.00	0.00	0.01	0.99	0.00	0.00	0.00
III,28	28	28	0.00	0.00	0.00	0.00	0.00	0.00	0.00	0.00	0.00	0.00	0.00	0.00	0.00	0.91	0.00	0.09
III,30	30	30	0.00	0.00	0.00	0.00	0.00	0.00	0.00	0.00	0.00	0.00	0.00	0.00	0.00	0.00	1.00	0.00
III,32	32	32	0.00	0.00	0.00	0.00	0.00	0.00	0.00	0.00	0.00	0.00	0.00	0.00	0.00	0.00	0.00	1.00
IV,2	2	2	1.00	0.00	0.00	0.00	0.00	0.00	0.00	0.00	0.00	0.00	0.00	0.00	0.00	0.00	0.00	0.00
IV,4	4	4	0.00	1.00	0.00	0.00	0.00	0.00	0.00	0.00	0.00	0.00	0.00	0.00	0.00	0.00	0.00	0.00
IV,6	6	6	0.00	0.00	0.96	0.04	0.00	0.00	0.00	0.00	0.00	0.00	0.00	0.00	0.00	0.00	0.00	0.00
IV,4	8	8	0.00	0.00	0.07	0.93	0.00	0.00	0.00	0.00	0.00	0.00	0.00	0.00	0.00	0.00	0.00	0.00
IV,10	10	10	0.00	0.00	0.00	0.00	1.00	0.00	0.00	0.00	0.00	0.00	0.00	0.00	0.00	0.00	0.00	0.00
IV,12	12	12	0.00	0.00	0.00	0.00	0.00	1.00	0.00	0.00	0.00	0.00	0.00	0.00	0.00	0.00	0.00	0.00
IV,14	14	14	0.00	0.00	0.00	0.00	0.00	0.00	1.00	0.00	0.00	0.00	0.00	0.00	0.00	0.00	0.00	0.00
IV,16	16	16	0.00	0.00	0.00	0.00	0.00	0.00	0.00	1.00	0.00	0.00	0.00	0.00	0.00	0.00	0.00	0.00
IV,18	18	18	0.00	0.00	0.00	0.00	0.00	0.00	0.00	0.00	1.00	0.00	0.00	0.00	0.00	0.00	0.00	0.00
IV,20	20	20	0.00	0.00	0.00	0.00	0.00	0.00	0.00	0.00	0.00	1.00	0.00	0.00	0.00	0.00	0.00	0.00
IV,22	22	22	0.00	0.00	0.00	0.00	0.00	0.00	0.00	0.00	0.00	0.00	1.00	0.00	0.00	0.00	0.00	0.00
IV,24	24	24	0.00	0.00	0.00	0.00	0.00	0.00	0.00	0.00	0.00	0.00	0.00	1.00	0.00	0.00	0.00	0.00
IV,26	26	26	0.00	0.00	0.00	0.00	0.00	0.00	0.00	0.00	0.00	0.00	0.00	0.00	1.00	0.00	0.00	0.00
IV,28	28	28	0.00	0.00	0.00	0.00	0.00	0.00	0.00	0.00	0.00	0.00	0.00	0.00	0.00	1.00	0.00	0.00
IV,30	30	30	0.00	0.00	0.00	0.00	0.00	0.00	0.00	0.00	0.00	0.00	0.00	0.00	0.00	0.00	1.00	0.00
IV,32	32	32	0.00	0.00	0.00	0.00	0.00	0.00	0.00	0.00	0.00	0.00	0.00	0.00	0.00	0.00	0.00	1.00

a Sample expressed as R,W: Region, maturation time in weeks, samples marked with * were misclassified.

## 2 Experimental design, materials, and methods

### 2.1 Tequila samples

**Fig. 1:**
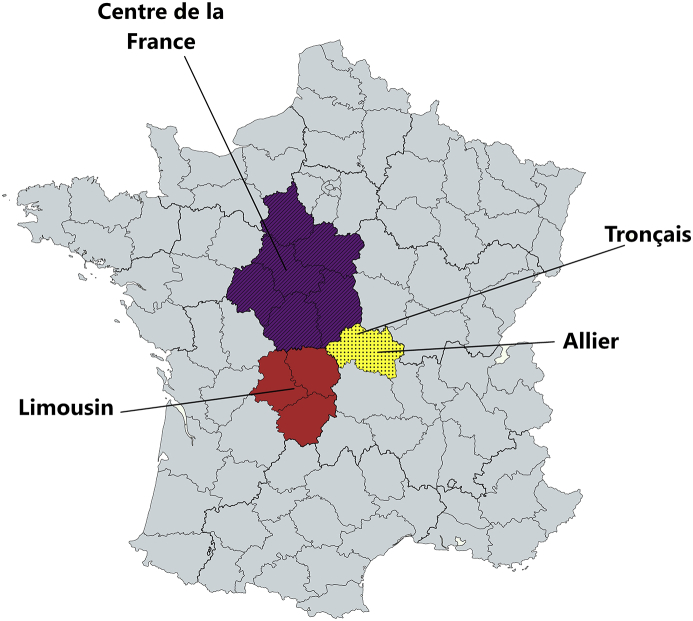
French barrels origin regions. Region: I, Allier; II, Limousin; III, Tronçais; IV, Centre de la France.

Tequila samples were obtained as described before [2]. One batch of 100% Agave Silver tequila directly obtained from distillation stage, 40% alcohol v/v, provided by a Tequila company (Jalisco, México) was placed in new oak barrels with a capacity of 230–240 L of 4 different French regions: Allier (Region I, RI), Limousin (Region II, RII), Tronçais (Region III, RIII), and Centre de la France (Region IV, RIV) (Fig. 1). All the oak barrels were made with the same cooperage method and were conditioned at the origin with medium intensity toasting (no more data were provided for the distillery). Four barrels from each region (n = 16) were filled with the tequila and maturated in the Tequila's company cellar under darkness (18 ± 5 °C and 50 ± 5% of relative humidity). Then, a 1 L sample was obtained from each barrel every 2 weeks until a final time of 32 weeks. Samples for each region at each sampling point were homogenized given one batch of 4 L for each region and sampling point. This method was selected instead of having 4 × 1 L samples since in Tequila's distilleries tequila from barrels filled with the same batch is mixed to obtain a homogeneous final product.

### 2.2 Volatile compounds analysis

Minor volatile compounds were extracted according to the methodology reported before [3]. First, the sample's alcohol content was adjusted to 30 mL of ethanol/100 mL by adding distilled water and ethanol content was verified with calibrated alcoholometer (Dujardin-Salleron, Paris) with the Gay-Lussac scale at 15 °C. Then 0.2 g of NaCl (A.C.S. J.T. Baker, Phillipsburg, NJ) was added to 325 mL of the alcohol adjusted sample and volatiles were extracted with 45 mL of a mixture of pentane/dichloromethane 3:1 (v/v) (HPLC, Fisher, Leicester, U.K.). After 5 min agitation, was let to stand until organic layer complete separation. The organic layer was recovered and dried with anhydrous Na_2_SO_4_ (Mallinckrodt, Paris, USA). Extracts were concentrated in a Kuderna-Danish device until a final volume of 0.4 mL, placed in suitable tight closed vials and kept at −40 °C until analysis. All the samples were extracted twice.

### 2.3 Gas chromatographic analysis (GC-MS)

Extracts were analyzed twice by gas chromatography in a gas chromatograph HP 5890 Series II (Hewlett-Packard, Palo Alto, USA) coupled to a mass detector (HP 5972), with a capillary DB-Wax polyethylene glycol column (30 m × 0.25 mm ID × 0.25 mm thickness, Hewlett-Packard). The oven program was 40 °C, 5 min, then increased at 2.5 °C/min until 220 °C and held for 35 min. Injector and detector temperatures were kept at 220 °C and 260 °C, respectively. A sample volume of 0.5 mL was automatically injected using helium as carrier gas at 0.8 mL/min and a 60:1 split ratio was used. The total ion chromatograms (TIC), as well as the mass spectra, were acquired in the electron impact (EI) mode at 70 eV and traced at 1.6 scans/s.

Compounds were tentatively identified by comparing the spectrum of each compound with the Wiley 175L spectra library. Identity was confirmed by comparing with reference standards (Sigma-Aldrich, St. Louis, MO, USA) with a purity >98% and/or by comparison with the Kovats index reported in the literature and by consulting bibliographical references of tequila and other spirits whose volatile composition has been studied in columns similar to the one used in the methodology of this study.

The quantification was performed with the percent area quantification method after subtraction of solvents area. This method was selected because chromatograms were too complex to add an internal standard.

### 2.4 Statistical analysis

The obtained dataset was analyzed using STATISTICA v13 (TIBCO Software Inc, USA). General Discriminant Analysis (GDA) was applied to the obtained datasets (Table 2, Table 3) to identify the compounds that made it possible to discriminate tequila samples according to the barrel origin region and to the maturation time. Forward or backward stepwise methods (*p* inclusion 0.05, *p* exclusion 0.05) were applied with the purpose of minimize the model size. The selected variables were those with a significant (*p* < 0.05) F value.
